# Impacts of climate change on aquatic insects in temperate alpine regions: Complementary modeling approaches applied to Swiss rivers

**DOI:** 10.1111/gcb.15637

**Published:** 2021-05-20

**Authors:** Pablo Timoner, Marc Fasel, Seyed Saeid Ashraf Vaghefi, Pierre Marle, Emmanuel Castella, Frédéric Moser, Anthony Lehmann

**Affiliations:** ^1^ enviroSPACE Group Department F.‐A. Forel for Environmental and Aquatic Sciences University of Geneva Institute for Environmental Sciences Geneva Switzerland; ^2^ Eawag Swiss Federal Institute of Aquatic Science and Technology Duebendorf Switzerland; ^3^ Aquatic Ecology Group Department F.‐A. Forel for Environmental and Aquatic Sciences University of Geneva Institute for Environmental Sciences Geneva Switzerland; ^4^ GRID‐Geneva University of Geneva Institute for Environmental Sciences Geneva Switzerland

**Keywords:** macroecological models, species distribution models, species richness, stacked species distribution models, stream macroinvertebrates, Switzerland

## Abstract

Freshwater biodiversity loss is a major concern, and global warming is already playing a significant role in species extinctions. Our main goal was to predict climate change impacts on aquatic insect species distribution and richness in Swiss running waters according to two climate change scenarios (RCP2.6 and RCP8.5), using different modeling approaches, that is, species distribution models (SDMs), stacked‐SDMs (S‐SDMs) and a macroecological model (MEM). We analyzed 10,808 reaches, used as spatial units for model predictions, for a total river network length of 20,610 km. Results were assessed at both the countrywide and the biogeographic regional scales. We used incidence data of 41 species of Ephemeroptera, Plecoptera and Trichoptera (EPT) from 259 sites distributed across Switzerland. We integrated a coupled model for hydrology and glacier retreat to simulate monthly time‐step discharge from which we derived hydrological variables. These, along with thermal, land‐cover, topographic and spatially explicit data, served as predictors for our ecological models. Predictions of occurrence probabilities and EPT richness were compared among the different regions, periods and scenarios. A Shiny web application was developed to interactively explore all the models’ details, to ensure transparency and promote the sharing of information. MEM and S‐SDMs approaches consistently showed that overall, climate change is likely to reduce EPT richness. Decrease could be around 10% in the least conservative scenario, depending on the region. Global warming was shown to represent a threat to species from high elevation, but in terms of species richness, running waters from lowlands and medium elevation seemed more vulnerable. Finally, our results suggested that the effects of anthropogenic activities could overweight natural factors in shaping the future of river biodiversity.

## Graphical abstract

Prediction of climate change impacts on Ephemeroptera, Plecoptera and Trichoptera (EPT) species distribution and richness in Swiss rivers and streams, using different modeling approaches, i.e. species distribution models (SDMs), stacked‐SDMs (S‐SDMs) and a macroecological model (MEM). MEM and S‐SDMs approaches consistently showed that climate change is likely to reduce EPT richness. Decrease could be around 10% in the least conservative scenario, depending on the region. Global warming was shown to represent a threat to species from high elevation, but in terms of species richness, running waters from lowlands and medium elevation seemed more vulnerable.

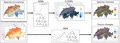

## 1 INTRODUCTION

Freshwater biodiversity loss represents a major concern (Reid et al., 2019; Strayer & Dudgeon, 2010). Species extinctions are indeed more frequent in freshwater than in other habitats (Wiens, 2016), and they are due to multiple causes, including global warming. The interactions of increased temperature and changes in sediment, nutrient and pollutant loadings can be limited through management and adaptation, yet direct impacts of climate change (i.e., increased temperature and changes in flow regimes) will hardly be prevented. As water temperature and flow regime are known to be primary drivers of freshwater species distribution (Bunn & Arthington, 2002; Ward & Stanford, 1982), changes in biodiversity of rivers and streams are expected. Beyond the question of whether species richness should be considered as something valuable per se, it has been shown that higher biodiversity leads to better ecosystem functions and higher delivery of ecosystem services (Grizzetti et al., 2019). From a conservation perspective, anticipating these changes is thus of high importance.

In response to the challenge of predicting streamflow at the catchment scale, various hydrological modeling tools have been developed (e.g., WaSiM Schulla, 1997; PREVAH Viviroli et al., 2009; SEHR‐ECHO Schaefli et al., 2014). One of them, the Soil and Water Assessment Tool (SWAT; Arnold et al., 1998), has been applied worldwide (Faramarzi et al., 2009; Rouholahnejad et al., 2014; Schuol et al., 2008), and simulated discharges from SWAT have already been used to assess species response to climate change (Sultana et al., 2020; Van Compernolle et al., 2019). However, to our knowledge, no coupled model for hydrology and glacier retreat has been integrated into ecological models yet.

Being relatively easy to identify and sensitive to environmental changes, aquatic insects belonging to the orders of Ephemeroptera, Plecoptera and Trichoptera (EPT) can be used as bioindicators of river and stream health (Lenat, 1988; Rosenberg & Resh, 1993). In running waters, high EPT richness (EPTr) is often considered as a proxy for good ecological status (Barbour et al., 1999). Behind this metric lays the assumption that in general, taxonomical diversity relates to functional diversity and integrity (De Arruda Almeida et al., 2018; Li et al., 2019).

Kaelin and Altermatt (2016) predicted α‐diversity (i.e., species richness at the local scale) of EPT species in the Swiss river network through a macroecological model (MEM; model that links a community‐level response like species richness to environmental variables), using landscape‐level predictors. Timoner et al. (2020) analyzed the effects of global warming on EPT composition at the same scale, but they focused on β‐diversity (i.e., assemblage diversity), and they did not consider changes in flow regimes. In both cases, preference was given to the “assemble first, predict later” approach (Ferrier et al., 2002; Ferrier & Guisan, 2006), with few or no insight into individual species. This procedure follows Clements (1916) by considering the community as an entity. On the contrary, the “predict first, assemble later” approach is based on Gleason's theory (Gleason, 1926) and considers that assemblages are made of a set of species that share, at least partially, the same environmental requirements. From a modeling perspective, this latter approach consists of the combination of individual species distribution models (SDMs), providing information at both the community and the species levels (e.g., Lehmann et al., 2002; Parviainen et al., 2009; Pineda & Lobo, 2009). Underlying assumptions about the ecological processes that control community assembly are different for both MEM and stacked SDMs (S‐SDMs). This means that the same predictors could reflect distinct causal processes (Dubuis et al., 2011). For instance, temperature might be related to the amount of energy available to the community in MEM, but would express a limitation for growth of single species in SDMs. However, the two approaches have shown to be complementary (D’Amen et al., 2015; Dubuis et al., 2011) and even congruent (Distler et al., 2015) to predict α‐diversity.

SDMs are widely used in ecological studies for predicting the spatial distribution of species (i.e., projections of realized niches; Franklin & Miller, 2010; Peterson et al., 2012; Guisan et al., 2017), representing the most popular modeling framework to project future range shifts in the face of global change (Ferrier et al., 2016). Various statistical techniques have been developed to describe the distributions of species in relation to environmental parameters. However, independent evaluations of models showed that no technique is fundamentally superior to the others (Olden & Jackson, 2002; Segurado & Araújo, 2004). Variability in forecasts depending on the chosen method challenges the practice of relying on one single technique to predict responses of species to climate change, but significant improvements on the robustness of predictions can be achieved with an ensemble approach (Araújo & New, 2007).

Previous studies carried out at national levels (i.e., Hamilton et al., 2010 for the USA; Li et al., 2013 for South Korea) quantified a major decrease in EPTr (~40%–50%) due to global warming by mid‐century. Our main goal here was to assess climate change impacts on EPT in Switzerland, by mid‐ and end‐century. We used forecasted hydrological and thermal data, according to two different climate scenarios (RCP2.6 and RCP8.5). We aimed at modeling the biodiversity from both the individual species and the species richness perspectives through an ensemble modeling technique and using different approaches (i.e., SDMs, S‐SDMs and MEM). While climate change could act as a major cause of biodiversity loss and species extinctions (Nunez et al., 2019; Thomas et al., 2004), it could also potentially increase species richness in cold regions (Hawkins et al., 2003). Therefore, we expected to observe an increase in EPTr at high altitude and conversely, a decrease at low altitude. This study provides new insights into the relative importance of climate change for the risk of biodiversity loss in running waters, and it may inform freshwater conservation planning in temperate alpine regions.

## 2 METHODS

### 2.1 Study area

Our study focused on rivers and streams throughout Switzerland (41,285 km^2^). The country shows a steep altitudinal gradient (range: 193 m a.s.l.–4634 m a.s.l.) with contrasted environmental conditions. We analyzed 10,808 reaches (median length of 1.47 km) modeled through SWAT and derived from the Swiss hydrographic network (total network length = 20,610 km; scale = 1:200,000; OFEV, 2018). They were used as spatial units for model predictions. Results were assessed at both the country and biogeographic regional scales (i.e., Jura, Plateau, Northern Alps, Central Eastern Alps, Southern Alps, Central Western Alps; Gonseth et al., 2001). The climate of the northern regions (i.e., Jura, Plateau and Northern Alps) is heavily influenced by the Atlantic Ocean. Winters in the plateau are mild and damp, whereas higher altitudes experience arctic temperatures. Conditions in the Central Alps are mostly intra‐alpine dry and continental, and the Southern Alps are mainly affected by the Mediterranean Sea, with relatively mild and dry winters and warm‐humid summers (MeteoSwiss, 2020). Regarding geology, the Plateau is dominated by unconsolidated rocks, the Jura and the Northern Alps by sedimentary rocks, and the Southern Alps by crystalline rocks (Swisstopo—Swiss Geotechnical Commission, 2006).

### 2.2 Species data

**FIGURE 1:**
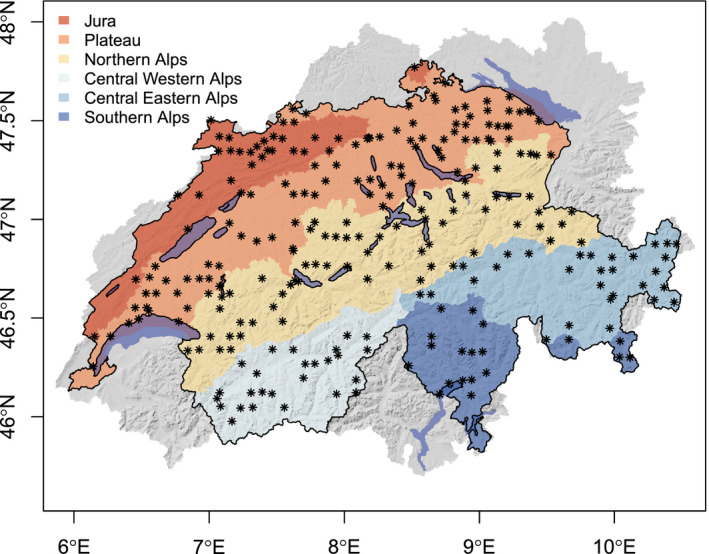
Location of the record sites used for model training. The different colors represent the different Swiss biogeographic regions. Gray color represents the areas located outside Switzerland used in the SWAT model and whose waters flow toward Swiss rivers

Species data were obtained from the Swiss Centre for the Cartography of the Fauna (InfoFauna—CSCF). Larvae of EPT were sampled between 2010 and 2017 in the framework of the Swiss Biodiversity Monitoring Program (BDM) and the National Surface Water Quality Monitoring Program (NAWA). Standard sampling method is described in Stucki (2019). We retained the sites that fell into the rivers and streams for which modeled discharge was available (SWAT stream network; distance tolerance: 30 m). To reduce potential false absences (i.e., a species is recorded as being absent in a study site, although it is present), which could increase SDMs bias, surveys where the number of identified species represented less than 70% of the minimum potential EPTr were discarded. This minimum potential EPTr was estimated by summing up the number of identified species, the number of identified genus that did not include any of the identified species, and the number of identified families that did not include any of the identified genus. When multiple surveys were available for the same site, we only considered the one with the highest percentage of identified species to avoid temporal autocorrelation issues. For modeling reasons, we focused on species with at least 25 occurrences (Guisan et al., 2017). The final sampling dataset included 259 sites (Figure 1) and 41 species (Table 1). Distribution of species elevation preferences was similar for both the selected species and the entire species pool (i.e., including rare species), making the difference between EPTr based on both sets of species relatively constant along the altitudinal gradient (−3.5 ± 2.5; mean ± SD).

**TABLE 1:** Species including their taxonomical information (Order and Family), their prevalence and their codes used in this study

Species	Order	Family	Prevalence	Code
*Allogamus auricollis* (Pictet, 1834)	Trichoptera	Limnephilidae	0.47	All.aur
*Amphinemura sulcicollis* (Stephens, 1836)	Plecoptera	Nemouridae	0.25	Amp.sul
*Baetis alpinus* (Pictet, 1843)	Ephemeroptera	Baetidae	0.66	Bae.alp
*Baetis lutheri* Müller‐Liebenau, 1967	Ephemeroptera	Baetidae	0.28	Bae.lut
*Baetis muticus* (Linnaeus, 1758)	Ephemeroptera	Baetidae	0.40	Bae.mut
*Baetis rhodani* (Pictet, 1843)	Ephemeroptera	Baetidae	0.80	Bae.rho
*Brachyptera risi* (Morton, 1896)	Plecoptera	Taeniopterygidae	0.28	Bra.ris
*Capnioneura nemuroides* Ris, 1905	Plecoptera	Capniidae	0.13	Cap.nem
*Centroptilum luteolum* Müller, 1776	Ephemeroptera	Baetidae	0.15	Cen.lut
*Chloroperla susemicheli* Zwick, 1967	Plecoptera	Chloroperlidae	0.16	Chl.sus
*Drusus discolor* (Rambur, 1842)	Trichoptera	Limnephilidae	0.17	Dru.dis
*Ecdyonurus helveticus* Eaton, 1883	Ephemeroptera	Heptageniidae	0.35	Ecd.hel
*Ecdyonurus picteti* (Meyer‐Dür, 1864)	Ephemeroptera	Heptageniidae	0.17	Ecd.pic
*Ecdyonurus torrentis* Kimmins, 1942	Ephemeroptera	Heptageniidae	0.10	Ecd.tor
*Ecdyonurus venosus* (Fabricius, 1775)	Ephemeroptera	Heptageniidae	0.26	Ecd.ven
*Epeorus alpicola* (Eaton, 1871)	Ephemeroptera	Heptageniidae	0.17	Epe.alp
*Epeorus assimilis* Eaton, 1865	Ephemeroptera	Heptageniidae	0.22	Epe.ass
*Ephemera danica* Müller, 1764	Ephemeroptera	Ephemeridae	0.14	Eph.dan
*Habroleptoides confusa* Sartori & Jacob, 1986	Ephemeroptera	Leptophlebiidae	0.24	Hab.con
*Habrophlebia lauta* McLachlan, 1884	Ephemeroptera	Leptophlebiidae	0.15	Hab.lau
*Halesus digitatus* (von Paula Schrank, 1781)	Trichoptera	Limnephilidae	0.26	Hal.dig
*Hydropsyche siltalai* Doehler, 1963	Trichoptera	Hydropsychidae	0.23	Hyd.sil
*Isoperla grammatica* (Poda, 1761)	Plecoptera	Perlodidae	0.16	Iso.gra
*Isoperla rivulorum* (Pictet & F.J., 1841)	Plecoptera	Perlodidae	0.10	Iso.riv
*Nemoura minima* Aubert, 1946	Plecoptera	Nemouridae	0.17	Nem.min
*Nemoura mortoni* Ris, 1902	Plecoptera	Nemouridae	0.34	Nem.mor
*Odontocerum albicorne* (Scopoli, 1763)	Trichoptera	Odontoceridae	0.20	Odo.alb
*Paraleptophlebia submarginata* (Stephens, 1835)	Ephemeroptera	Leptophlebiidae	0.17	Par.sub
*Perla grandis* Rambur, 1842	Plecoptera	Perlidae	0.12	Per.gra
*Philopotamus ludificatus* McLachlan, 1878	Trichoptera	Philopotamidae	0.10	Phi.lud
*Potamophylax cingulatus* (Stephens, 1837)	Trichoptera	Limnephilidae	0.30	Pot.cin
*Protonemura brevistyla* (Ris, 1902)	Plecoptera	Nemouridae	0.13	Pro.bre
*Protonemura lateralis* (Pictet & F.J., 1836)	Plecoptera	Nemouridae	0.29	Pro.lat
*Protonemura nimborum* (Ris, 1902)	Plecoptera	Nemouridae	0.10	Pro.nim
*Rhithrogena alpestris* Eaton, 1885	Ephemeroptera	Heptageniidae	0.19	Rhi.alp
*Rhithrogena loyolaea* Navás, 1922	Ephemeroptera	Heptageniidae	0.14	Rhi.loy
*Rhithrogena puthzi* Sowa, 1984	Ephemeroptera	Heptageniidae	0.20	Rhi.put
*Rhithrogena semicolorata* (Curtis, 1834)	Ephemeroptera	Heptageniidae	0.34	Rhi.sem
*Rhyacophila torrentium* Pictet, 1834	Trichoptera	Rhyacophilidae	0.17	Rhy.tor
*Rhyacophila tristis* Pictet, 1834	Trichoptera	Rhyacophilidae	0.41	Rhy.tri
*Serratella ignita* (Poda, 1761)	Ephemeroptera	Ephemerellidae	0.12	Ser.ign

### 2.3 Model predictors

We used hydrological, thermal, land‐cover, topographic and spatial variables as predictors of EPT species distribution and richness.

Average monthly discharge of stream was simulated using SWAT. The entire SWAT stream network was derived from the Swiss hydrographic network (OFEV, 2018), and the European catchments and the Rivers network system (Ecrins; European Environment Agency, 2018) for tributaries of Swiss rivers that are outside Switzerland. SWAT model inputs included climatic data from 471 meteorological stations (Agenzia Regionale per la Protezione Ambientale, 2018; Météo France, 2018; MeteoSwiss, 2018b), soil information (1 km resolution; Food & Agriculture Organization, 2012), land‐cover data (European Environment Agency, 2019; WSL, 2018; 100 m), digital elevation map (European Environment Agency, 2017; Swisstopo, 2018; 25 m), and data from agricultural management practices (Swiss Farmers’ Union, 2018). Flow discharge data from 91 gauging stations distributed across the entire country served for SWAT model calibration using the SWAT‐CUP tool (Abbaspour, 2014). For more details about the calculation of the water balance of the SWAT model, see Appendix S1. According to the percent bias measure (PBIAS) and the performance rating recommended by Moriasi et al. (2007), quality of discharge simulation was “very good,” “good” or “satisfactory” for 74% of the 91 gauging stations (for more details regarding PBIAS, see Appendix S2). A glacier evolution runoff model (GERM; Ashraf Vaghefi et al., 2019; Farinotti et al., 2012) was run for the major glaciers where downstream flow discharge data were available for the GERM calibration. Glacier melt time series were then added to the SWAT model. This accounted for the ice melt contribution in stream flow, at least for some of the largest glacier catchments (e.g., Aletsch, Lower Grindelwald, Rhône, Morteratsch, etc.).

Average monthly water temperature was predicted using average monthly air temperature and the interaction between the latter and the area percentage of glacier or lake cover in the catchments. Rasters of air temperature at 1 km resolution were obtained from MeteoSwiss (MeteoSwiss, 2018a), and land‐cover raster at 100 m resolution was obtained from the Corine Land Cover inventory (CLC2012; European Environment Agency, 2019; WSL, 2018). Actual water temperature data obtained between 2015 and 2017 at 69 stations of the water temperature monitoring network (OFEV, 2017) were used to fit a linear mixed model to account for repeated measures, allowing a random slope effect for air temperature. Fixed effects explained 84% of the variance (marginal *R* ^2^) while the entire model explained 92% (conditional *R* ^2^). See Table S3.1 and Figure S3.1 for the mixed model summary and diagnostic plots. Hydrological and thermal predictors of EPT species distribution and richness included the mean, or the log‐transformed mean in the case of the hydrological predictor, and the variation coefficient (i.e., standard deviation divided by the mean) of average monthly values. For model training, these predictors were calculated over the year preceding the sampling dates, as the majority of the sampled species have a life cycle duration shorter or equal to one year (Tachet et al., 2010). For predictions, yearly values (i.e., mean and variation coefficient) were averaged over 10‐year periods (i.e., 2015–2025, 2055–2065 and 2080–2090). Bias‐corrected climate change scenarios to calculate both the flow discharge and the water temperature were derived from climate scenarios for Switzerland “CH2018” (Feigenwinter et al., 2018), which originated from the EURO‐CORDEX initiative (Jacob et al., 2014; Kotlarski et al., 2014). Two climate models were retained for this study, namely the SMHI‐RCA‐ECEARTH‐EUR11 and the MPICSC‐REMO2‐MPIESM‐EUR44 (see Table S4.2 for more details). The former predicts a reduction in precipitation, whereas the latter predicts an increase (Zarrineh et al., 2020). Following an ensemble approach, data from both simulations were averaged. We considered two Representative Concentration Pathways (RCP), namely the RCP2.6 (the most conservative) and the RCP8.5 (the least conservative), labeled according to the approximate total radiative forcing level in year 2100 relative to year 1750 (2.6 and 8.5 W/m^2^, respectively). Glacier coverage, used as covariate to estimate the water temperature, was adapted for each period and pathway, based on Zekollari et al. (2019). The lowest 45% (RCP2.6) or 50% (RCP8.5) glacier pixels were “replaced” by bare rocks for the period 2055–2065, whereas the lowest 60% (RCP2.6) or 90% (RCP8.5) of them were replaced for the period 2080–2090. This approximate method relied on the assumption that glacier area is reducing from lower to higher elevations. Land‐cover variables comprised urban, forest and agriculture cover in the catchment. Slope used as proxy for flow velocity was derived from a digital elevation model using ArcGIS 10.5 (ESRI, 2017). Raster data were obtained from swisstopo (swissALTI^3^ *^D^*, 2 m resolution resampled to 25 m through a bilinear interpolation; Swisstopo, 2018). All environmental predictors are summarized in Table 2.

**TABLE 2:** Environmental variables used as predictors for EPT species distribution and richness models (WSL, Swiss Federal Institute for Forest, Snow and Landscape Research; EEA, European Environment Agency)

Predictor	Unit	Source
Mean water temperature	°C	Modeled in this study
Coefficient of variation of water temperature	—	Modeled in this study
Log‐transformed mean water discharge	m^3^/s	Modeled in this study
Coefficient of variation of water discharge	—	Modeled in this study
Slope	%	Federal Office of Topography
Urban cover	%	WSL and EEA
Forest cover	%	WSL and EEA
Agriculture cover	%	WSL and EEA

The magnitude to which the presence of spatial autocorrelation in the residuals becomes detrimental to the modeling process is still debated (Gaspard et al., 2019). However, spatially explicit models to control for it have been shown to be more realistic in the context of species’ range shift (Crase et al., 2014) or expansion (De Marco et al., 2008), or in conservation planning (Domisch et al., 2019). Thus, we opted to add the first five eigenvectors (i.e., large‐scale geographic patterns) extracted from a doubly centered Euclidean distance matrix (i.e., between reaches) as covariates (Diniz‐Filho & Bini, 2005; Dormann et al., 2007; Griffith & Peres‐Neto, 2006). As eigen decomposition is computationally demanding for large samples, we used a fast and robust approximation of eigenvectors (Murakami & Griffith, 2019).

All Pearson's correlation coefficients between predictors were lower than 0.7 and all variables had a variance inflation factor (VIF) lower than 4.

Spatial analyses were performed in R (R Core Team, 2019), using the following packages: “raster” (Hijmans, 2019), “rgdal” (Bivand et al., 2019), “rgeos” (Bivand & Rundel, 2019), “maptools” (Bivand & Lewin‐Koh, 2019), “igraph” (Csardi & Nepusz, 2006) and “sporman” (Murakami, 2020).

### 2.4 Modeling framework

SDMs were implemented by binomial generalized linear models (GLM; McCullagh & Nelder, 1989) and random forest classifiers (RF; Breiman, 2001), followed by an ensemble forecast (Marmion et al., 2009) weighted by the area under the receiver operating curve (AUC; Fielding & Bell, 1997). MEM was run by a Poisson GLM and a RF regressor, followed by an ensemble forecast weighted by the Spearman's correlation coefficient between observations and predictions. We used a “community cross‐validation” approach (CCV; Scherrer et al., 2018), using a repeated split‐sample procedure for models’ evaluation (*N* = 50; 80%–20%). Variable importance was assessed through a random permutation procedure (Thuiller et al., 2019), and response curves were obtained by an adaptation of the evaluation strip proposed by Elith et al. (2005) and Thuiller et al. (2019). For each predictor type (i.e., hydrological, thermal, land‐cover, topographic and spatial), the sum of importance values was divided by the number of variables to allow comparison between types. Both AUC and true skill statistic (TSS; Allouche et al., 2006) methods were used to assess the SDMs predictive performance. A value of AUC higher than 0.90 was considered to be excellent, 0.80–0.90 good, 0.70–0.80 fair and 0.60–0.70 poor (Swets, 1988). For TSS values, higher than 0.75 was considered to be excellent, 0.40–0.75 good and less than 0.40 poor (Landis & Koch, 1977). Predicted occurrence probabilities were compared among the different periods for each scenario to assess the potential impact of climate change on individual species.

SDMs were stacked by summing up the predicted probabilities of all species at each site, assuming that EPTr prediction follows a Poisson‐binomial distribution (Calabrese et al., 2014; Dubuis et al., 2011). Based on the probability mass function, we could accept or reject (α = 0.05) the null hypothesis that there was no difference between the predicted and observed EPTr (see Calabrese et al., 2014; Scherrer et al., 2020 for more details). Predictions from MEM and S‐SDMs were assessed by testing their linear relationship with observations (linear hypothesis test; α = 0.05). Predicted EPTr were compared between both MEM and S‐SDMs approaches, periods and climate change scenarios.

All models were run in R (R Core Team, 2019), using the “randomForest” package (Liaw & Wiener, 2002) for random forest models, the “pROC” package for calculating AUC (Robin et al., 2011), the “biomod2” package for calculating TSS (Thuiller et al., 2019), the “poisbinom” package (Olivella & Shiraito, 2017) for the implementation of the Poisson‐binomial distribution and the “car” package (Fox & Weisberg, 2019) for linear hypothesis assessment.

### 2.5 Shiny web application

To ensure transparency and promote the sharing of information, we developed a web application using the “Shiny” package (Chang et al., 2019), which allows an interactive exploration of all the models’ results and outputs (i.e., maps, predictor response curves, future projections, model performance, etc.). It can be accessed at http://lsshiny1.unige.ch:8080/swatch21bio/.

## 3 RESULTS

### 3.1 Species distribution models

**FIGURE 2:**
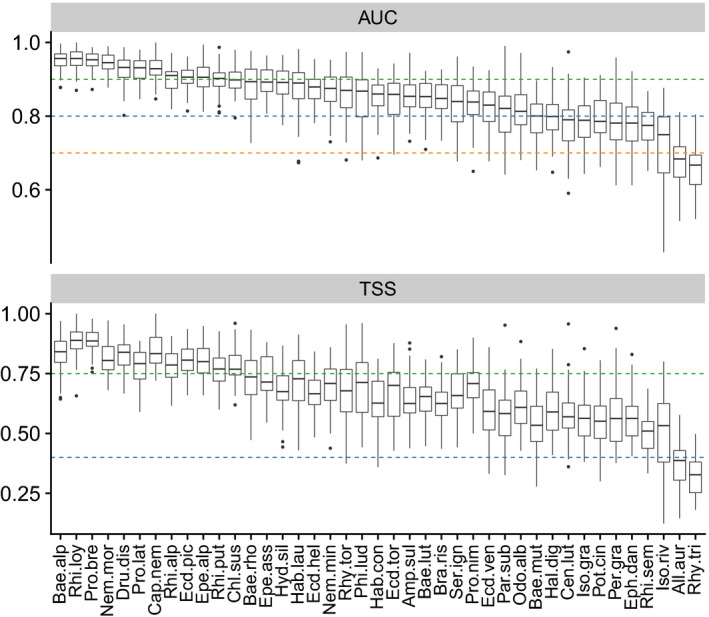
Distribution of AUC and TSS values for each species across 50 split samples. Taxa are ordered by their median AUC values. Green, blue and orange dashed lines represent thresholds for “excellent,” “good” and “fair” models. Boxplots show the median (horizontal line) and the interquartile range (IQR; box outline). The whiskers extend from the hinge to the highest and lowest value that are within 1.5*IQR of the hinge

**FIGURE 3:**
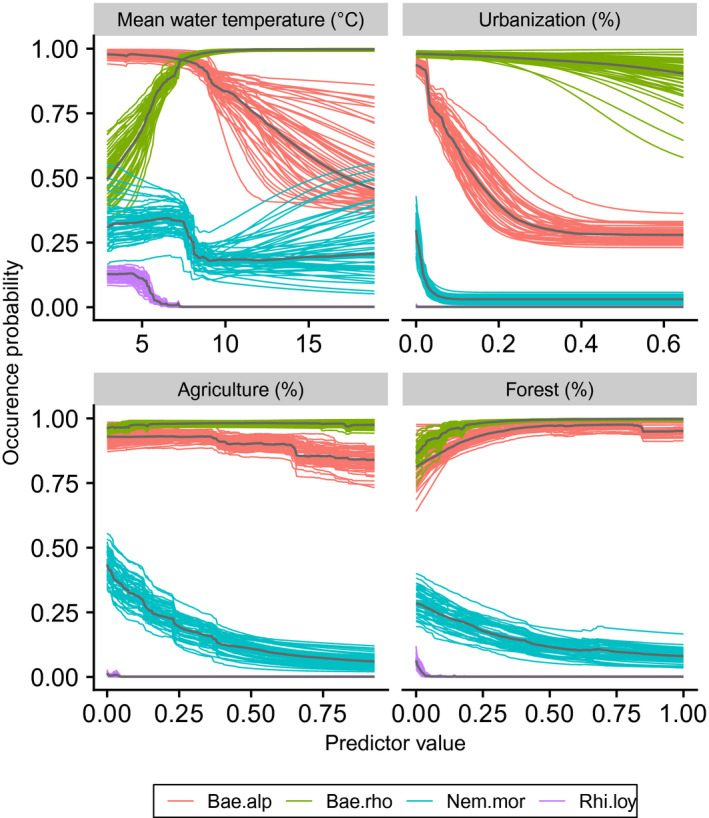
Occurrence probability along the four most important predictors for *Baetis alpinus*, *B*. *rhodani*, *Nemoura mortoni* and *Rhithrogena loyolaea*, calculated at each of the 50 split samples. Gray lines represent median response curves

**FIGURE 4:**
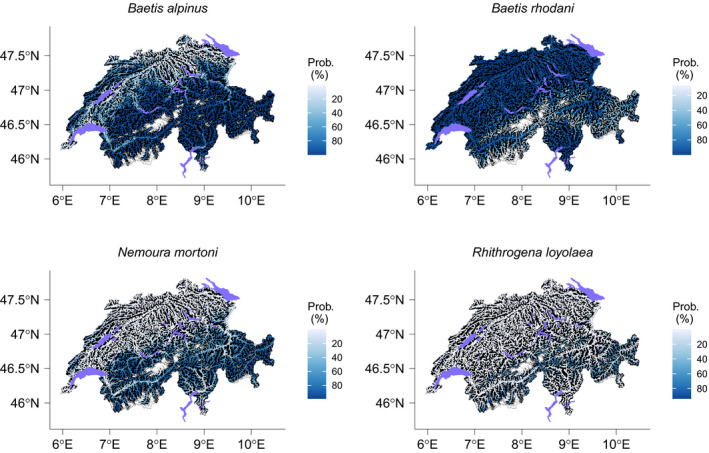
Occurrence probability for *Baetis alpinus*, *B*. *rhodani*, *Nemoura mortoni* and *Rhithrogena loyolaea* in Switzerland. Lakes are in purple and gray areas represent glaciers

Median AUC and TSS of ensemble models across all species and split samples were 0.86 and 0.68, respectively. According to the AUC and considering each species separately, 29%, 49% and 17% of the models were considered as “excellent,” “good” and “fair,” respectively (Figure 2). Two species models (*Rhyacophila tristis* and *Allogamus auricollis*) resulted to be “poor.” According to the TSS, 29% and 66% of the models were regarded as “excellent” and “good,” respectively (Figure 2), and the same above‐mentioned models (*R*. *tristis* and *A*. *auricollis*) were also considered as “poor.” Variable importance varied among taxa, but land‐cover outperformed other types of predictors to explain species distribution for 85% of the taxa. Over all species, the standardized sum of land‐cover importance values was 44%, 61%, 78% and 58% higher than for thermal, hydrological, topographic and spatial predictors, respectively. As expected, response curves and distribution varied among species, even from the same genus (Figures 3 and 4).

Relative changes in mean occurrence probability at the national scale between 2015–2025 and 2080–2090 for scenario RCP2.6 varied from −8.2% to +5.7% with a median relative change of −0.2% (Table 3). For scenario RCP8.5, they varied from −52.1% to +86.5%, with a median value of −5.1% (Table 4). For more details about changes in occurrence probability in the different Swiss biogeographic regions, see Appendix S5.

**TABLE 3:** Changes in mean occurrence probability ($\bar{p}$) of species according to scenario RCP2.6 in all of Switzerland. Relative change (%) is calculated with respect to period 2015–2025. Species are ordered according to the relative change in 2080–2090

Species	2015–2025	2055–2065		2080–2090	
	$\bar{p}$	$\bar{p}$	Δ (%)	$\bar{p}$	Δ (%)
*Rhithrogena loyolaea*	0.0940	0.0846	−10.0%	0.0862	−8.2%
*Protonemura brevistyla*	0.0896	0.0846	−5.6%	0.0849	−5.2%
*Nemoura minima*	0.1900	0.1791	−5.8%	0.1817	−4.4%
*Capnioneura nemuroides*	0.1448	0.1376	−5.0%	0.1391	−3.9%
*Serratella ignita*	0.1448	0.1450	+0.1%	0.1395	−3.7%
*Rhyacophila tristis*	0.4437	0.4294	−3.2%	0.4346	−2.0%
*Allogamus auricollis*	0.4800	0.4714	−1.8%	0.4716	−1.8%
*Rhithrogena puthzi*	0.1809	0.1743	−3.6%	0.1778	−1.7%
*Brachyptera risi*	0.3079	0.3025	−1.8%	0.3034	−1.5%
*Habrophlebia lauta*	0.1373	0.1300	−5.3%	0.1352	−1.5%
*Protonemura nimborum*	0.0839	0.0815	−2.9%	0.0829	−1.3%
*Halesus digitatus*	0.2620	0.2556	−2.5%	0.2589	−1.2%
*Odontocerum albicorne*	0.2252	0.2224	−1.2%	0.2227	−1.1%
*Baetis lutheri*	0.2680	0.2666	−0.5%	0.2656	−0.9%
*Isoperla grammatica*	0.1794	0.1785	−0.5%	0.1780	−0.8%
*Perla grandis*	0.1375	0.1357	−1.3%	0.1364	−0.8%
*Potamophylax cingulatus*	0.3117	0.3105	−0.4%	0.3092	−0.8%
*Amphinemura sulcicollis*	0.2854	0.2834	−0.7%	0.2834	−0.7%
*Baetis alpinus*	0.6667	0.6578	−1.3%	0.6625	−0.6%
*Rhithrogena semicolorata*	0.3759	0.3749	−0.3%	0.3748	−0.3%
*Baetis muticus*	0.4288	0.4272	−0.4%	0.4279	−0.2%
*Centroptilum luteolum*	0.1444	0.1430	−1.0%	0.1441	−0.2%
*Ecdyonurus venosus*	0.2512	0.2493	−0.7%	0.2508	−0.2%
*Ecdyonurus picteti*	0.1533	0.1508	−1.7%	0.1532	−0.1%
*Nemoura mortoni*	0.3181	0.3138	−1.3%	0.3185	+0.1%
*Chloroperla susemicheli*	0.1348	0.1333	−1.1%	0.1353	+0.3%
*Rhithrogena alpestris*	0.1683	0.1663	−1.2%	0.1690	+0.4%
*Habroleptoides confusa*	0.2450	0.2437	−0.5%	0.2463	+0.5%
*Drusus discolor*	0.1194	0.1165	−2.4%	0.1201	+0.6%
*Ecdyonurus helveticus*	0.3697	0.3699	0.0%	0.3727	+0.8%
*Epeorus assimilis*	0.2470	0.2499	+1.2%	0.2491	+0.8%
*Baetis rhodani*	0.8386	0.8494	+1.3%	0.8481	+1.1%
*Hydropsyche siltalai*	0.2005	0.2061	+2.8%	0.2032	+1.3%
*Paraleptophlebia submarginata*	0.1554	0.1562	+0.5%	0.1575	+1.3%
*Protonemura lateralis*	0.2696	0.2708	+0.4%	0.2734	+1.4%
*Rhyacophila torrentium*	0.1942	0.1970	+1.4%	0.1973	+1.6%
*Epeorus alpicola*	0.1352	0.1341	−0.8%	0.1379	+2.0%
*Isoperla rivulorum*	0.0933	0.0952	+2.0%	0.0956	+2.4%
*Ecdyonurus torrentis*	0.0709	0.0696	−1.8%	0.0727	+2.6%
*Ephemera danica*	0.1214	0.1265	+4.2%	0.1255	+3.4%
*Philopotamus ludificatus*	0.0930	0.0991	+6.6%	0.0983	+5.7%

**TABLE 4:** Changes in mean occurrence probability ($\bar{p}$) of species according to scenario RCP8.5 in all of Switzerland. Relative change (%) is calculated with respect to period 2015–2025. Species are ordered according to the relative change in 2080–2090

Species	2015–2025	2055–2065		2080–2090	
	$\bar{p}$	$\bar{p}$	Δ (%)	$\bar{p}$	Δ (%)
*Rhithrogena loyolaea*	0.0918	0.0655	−28.6%	0.0440	−52.1%
*Protonemura brevistyla*	0.0886	0.0693	−21.8%	0.0515	−41.9%
*Rhithrogena puthzi*	0.1794	0.1492	−16.8%	0.1216	−32.2%
*Rhithrogena alpestris*	0.1685	0.1410	−16.3%	0.1167	−30.8%
*Protonemura nimborum*	0.0833	0.0704	−15.5%	0.0607	−27.2%
*Rhyacophila tristis*	0.4437	0.3828	−13.7%	0.3264	−26.4%
*Ecdyonurus picteti*	0.1525	0.1308	−14.2%	0.1124	−26.3%
*Drusus discolor*	0.1182	0.1019	−13.8%	0.0880	−25.6%
*Habrophlebia lauta*	0.1343	0.1089	−18.9%	0.1062	−20.9%
*Nemoura minima*	0.1840	0.1657	−9.9%	0.1510	−17.9%
*Nemoura mortoni*	0.3169	0.2902	−8.4%	0.2629	−17.1%
*Rhyacophila torrentium*	0.1949	0.1786	−8.4%	0.1615	−17.1%
*Baetis alpinus*	0.6686	0.6171	−7.7%	0.5673	−15.2%
*Brachyptera risi*	0.3063	0.2954	−3.6%	0.2687	−12.3%
*Ecdyonurus venosus*	0.2531	0.2381	−5.9%	0.2227	−12.0%
*Halesus digitatus*	0.2599	0.2396	−7.8%	0.2288	−11.9%
*Epeorus alpicola*	0.1348	0.1270	−5.8%	0.1220	−9.5%
*Capnioneura nemuroides*	0.1386	0.1329	−4.1%	0.1260	−9.1%
*Allogamus auricollis*	0.4750	0.4550	−4.2%	0.4413	−7.1%
*Ecdyonurus helveticus*	0.3717	0.3579	−3.7%	0.3466	−6.8%
*Ecdyonurus torrentis*	0.0702	0.0635	−9.6%	0.0667	−5.1%
*Baetis lutheri*	0.2661	0.2709	+1.8%	0.2555	−4.0%
*Isoperla grammatica*	0.1798	0.1768	−1.6%	0.1736	−3.4%
*Odontocerum albicorne*	0.2233	0.2198	−1.6%	0.2160	−3.3%
*Chloroperla susemicheli*	0.1340	0.1301	−2.9%	0.1297	−3.2%
*Amphinemura sulcicollis*	0.2848	0.2834	−0.5%	0.2761	−3.1%
*Rhithrogena semicolorata*	0.3744	0.3771	+0.7%	0.3631	−3.0%
*Isoperla rivulorum*	0.0935	0.0906	−3.1%	0.0923	−1.3%
*Protonemura lateralis*	0.2695	0.2714	+0.7%	0.2705	+0.4%
*Potamophylax cingulatus*	0.3117	0.3166	+1.6%	0.3168	+1.6%
*Habroleptoides confusa*	0.2448	0.2402	−1.9%	0.2500	+2.1%
*Perla grandis*	0.1356	0.1350	−0.5%	0.1389	+2.4%
*Baetis muticus*	0.4266	0.4370	+2.5%	0.4438	+4.1%
*Centroptilum luteolum*	0.1422	0.1415	−0.5%	0.1516	+6.6%
*Baetis rhodani*	0.8408	0.8779	+4.4%	0.9062	+7.8%
*Epeorus assimilis*	0.2502	0.2597	+3.8%	0.2770	+10.7%
*Paraleptophlebia submarginata*	0.1543	0.1617	+4.8%	0.1816	+17.7%
*Hydropsyche siltalai*	0.2047	0.2286	+11.7%	0.2535	+23.8%
*Serratella ignita*	0.1459	0.1706	+16.9%	0.1815	+24.5%
*Ephemera danica*	0.1199	0.1455	+21.4%	0.1859	+55.1%
*Philopotamus ludificatus*	0.0956	0.1307	+36.7%	0.1783	+86.5%

### 3.2 EPT richness models

**FIGURE 5:**
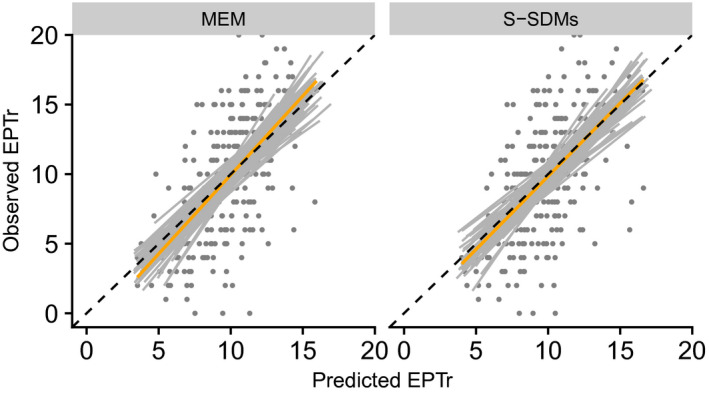
Predicted versus observed EPTr. Gray lines represent linear regressions for each split sample, the orange line is the linear regression calculated on median values and the dashed diagonal line reflects perfect linear relationship (i.e., x=y relationship)

Mean standardized EPTr error (i.e., the difference between prediction and observation standardized by the mean EPTr) across sites was approximately 0 in both MEM and S‐SDMs approaches (+0.01 ± 0.06 and +0.02 ± 0.06, respectively; mean ± SD). Linear hypothesis between observed and predicted EPTr was accepted in 90% (MEM) and 92% (S‐SDMs) of the split samples (Figure 5). Furthermore, based on S‐SDMs and the probability mass function of the Poisson‐binomial distribution, the percentage of sites with no significant difference between observation and prediction varied from 50% to 81% with a median value of 65%. At lower EPTr, the MEM approach tended to predict lower EPTr than S‐SDMs approach and vice versa, although differences were low (Figure 5). Overall, correlation between MEM and S‐SDMs predictions was 0.91.

**FIGURE 6:**
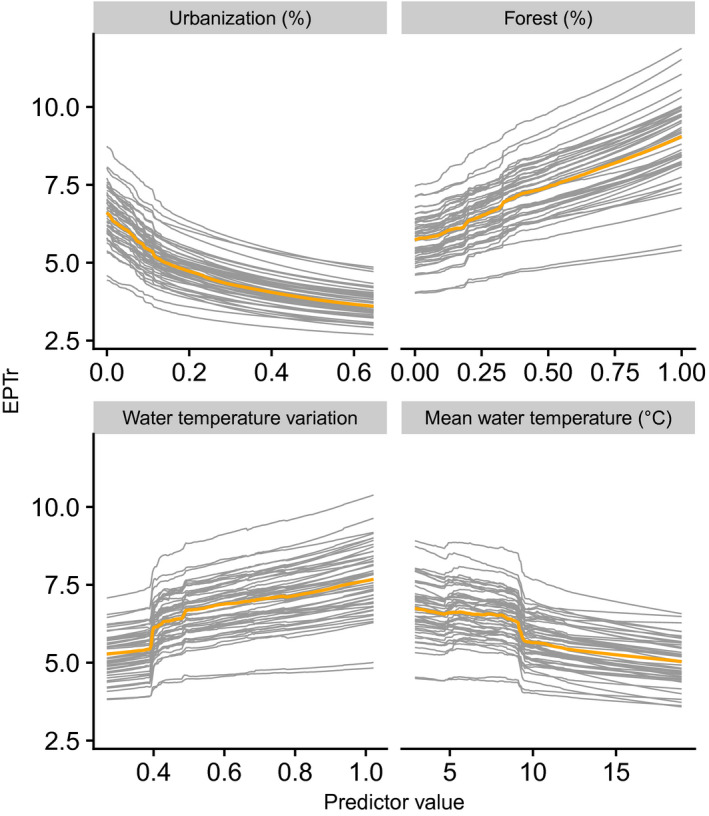
Response curves of EPTr along the four most important MEM predictors, calculated at each split sample (gray lines). Orange lines represent median response curves

The standardized sum of land‐cover importance values based on MEM was 35%, 86%, 93% and 97% higher than for thermal, hydrological, topographic and spatial predictors, respectively. EPTr clearly decreased with urbanization and increased with forest cover in the catchment. Moreover, it tended to be favored by relatively high annual water temperature variation and low mean water temperature (Figure 6).

Predicted EPTr was always lower in scenario RCP8.5 than in scenario RCP2.6 (Tables 5 and 6). Differences between RCP2.6 and RCP8.5 were approximately twice as important in 2080–2090 than in 2055–2065. The maximum disparity was found in Jura with a mean EPTr 11.6% lower in scenario RCP8.5 with respect to scenario RCP2.6 (Table 6). At the national scale, mean EPTr was 8.2% lower in scenario RCP8.5 with respect to scenario RCP2.6 for the period 2080–2090.

**TABLE 5:** Regional differences in mean predicted EPTr ($\bar{\text{E}P\text{Tr}}$), between methods (S‐SDMs versus MEM) and climate scenarios (RCP8.5 versus RCP2.6), for 2055–2065. The relative differences (Δ%) reflect the S‐SDMs and RCP8.5 $\bar{\text{E}P\text{Tr}}$ with respect to MEM and RCP2.6 $\bar{\text{E}P\text{Tr}}$, respectively

Region	S‐SDMs versus MEM		RCP8.5 versus RCP2.6	
	${\bar{\text{EPTr}}}_{\text{S - SDMs}}$	Δ%	${\bar{\text{EPTr}}}_{\text{RCP}8.5}$	Δ%
All Switzerland	9.6	+0.2%	9.4	−3.5%
Jura	10.7	+3.9%	10.2	−5.1%
Plateau	8.0	+5.6%	7.6	−4.7%
Northern Alps	10.9	−1.4%	10.7	−3.7%
Central Eastern Alps	10.4	−4.9%	10.5	−2.4%
Southern Alps	9.7	−2.1%	9.7	−1.9%
Central Western Alps	8.5	−2.5%	8.5	−1.9%

**TABLE 6:** Regional differences in mean predicted EPTr ($\bar{\text{E}P\text{Tr}}$), between methods (S‐SDMs versus MEM) and climate scenarios (RCP8.5 versus RCP2.6), for 2080–2090. The relative differences (Δ%) reflect the S‐SDMs and RCP8.5 $\bar{\text{E}P\text{Tr}}$ with respect to MEM and RCP2.6 $\bar{\text{E}P\text{Tr}}$, respectively

Region	S‐SDMs versus MEM		RCP8.5 versus RCP2.6	
	${\bar{\text{EPTr}}}_{\text{S - SDMs}}$	Δ%	${\bar{\text{EPTr}}}_{\text{RCP}8.5}$	Δ%
All Switzerland	9.5	+2.1%	9.0	−8.2%
Jura	10.5	+6.2%	9.6	−11.6%
Plateau	8.0	+7.6%	7.4	−9.0%
Northern Alps	10.7	+0.9%	10.1	−10.0%
Central Eastern Alps	10.3	−4.1%	10.2	−5.9%
Southern Alps	9.7	−0.4%	9.4	−6.0%
Central Western Alps	8.6	−1.2%	8.5	−2.6%

**FIGURE 7:**
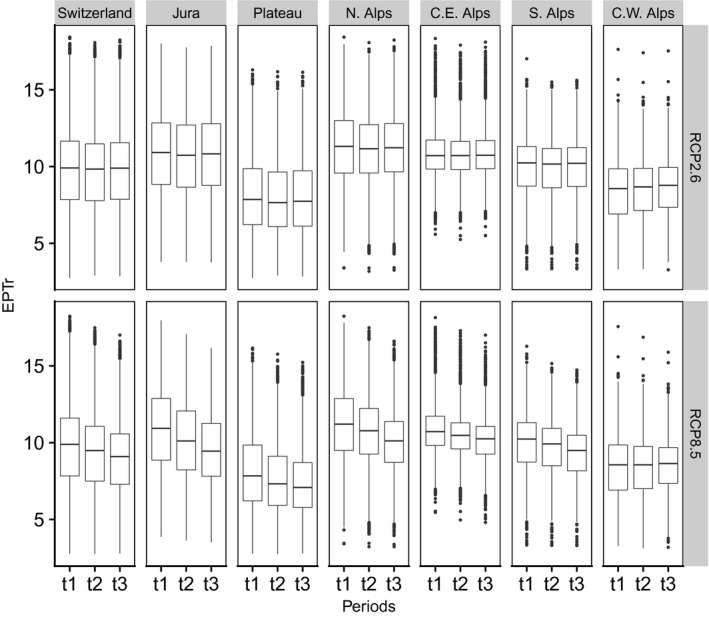
Distribution of EPT richness (EPTr) at the national scale and by region (N. = Norhtern; C.E. = Central Eastern; S. = Southern; C.W. Central Western) according to periods (t1 = 2015–2025; t2 = 2055–2065; t3 = 2080–2090) and climate change scenarios. For more details regarding boxplots, see Figure 1

**FIGURE 8:**
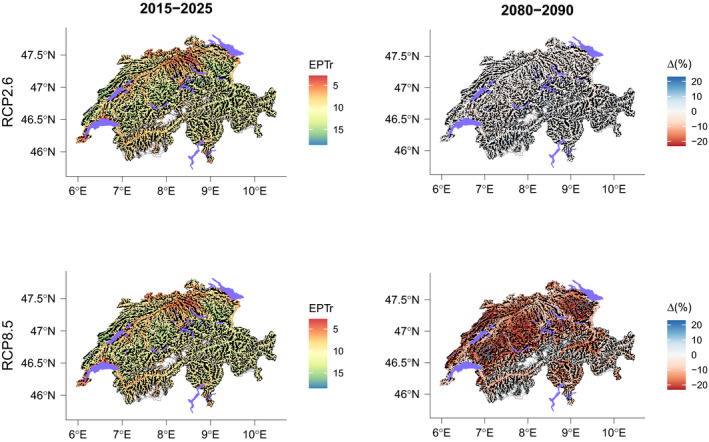
EPT richness (EPTr) based on MEM predictions in the Swiss river network for 2015–2025 and the relative projected changes for 2080–2090 according to two climate change scenarios

**FIGURE 9:**
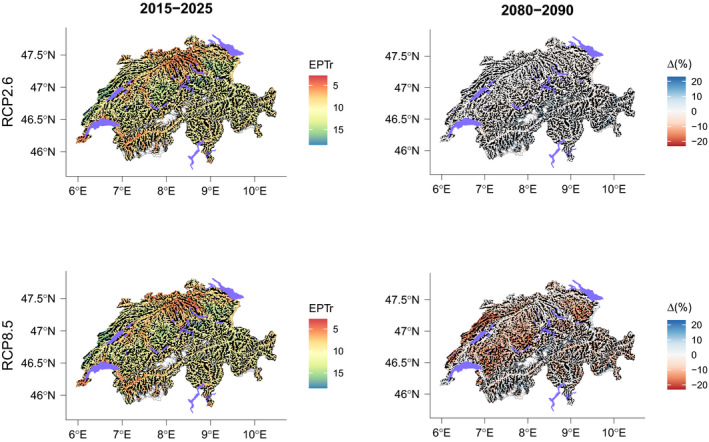
EPT richness (EPTr) based on S‐SDMs predictions in the Swiss river network for 2015–2025 and the relative projected changes for 2080–2090 according to two climate change scenarios

Changes in EPTr in scenario RCP2.6 were null or very low over time (Figures 7, 8, 9). However, we observed for some regions that EPTr slightly decreased by mid‐century and rose again by end‐century (e.g., Jura and Plateau; Figure 7). On the contrary, in scenario RCP8.5, changes were continuous with time and followed a marked decreasing trend, except in the Central Eastern and Western Alps.

## 4 DISCUSSION

Land‐cover outperformed other predictors in explaining EPT species distribution and richness in most of our models. We hypothesize that the spatial organization of human activities is relatively more important than natural factors in controlling biodiversity for Swiss rivers and streams. Because of the steep altitudinal gradient, land‐cover is indeed related to elevation and thus to other environmental variables like water temperature. This complicates causal interpretations, but the correlations among environmental variables were relatively low and the assessment of their relative contributions showed unambiguous results, which supports our hypothesis. This idea of human activity overweighting other macroecological processes in shaping communities has already been suggested (Munguía et al., 2016; Sebastián‐González et al., 2019).

Multiple studies have evidenced that land‐cover impacts freshwater species diversity through pollution and habitat degradation (Allan, 2004; Feld, 2013; García‐Vega & Newbold, 2020). Our results were in line with Paul and Meyer (2001) and Chamberlain et al. (2019), as they showed a clear negative relationship between urbanization and EPTr. At the national scale, the effect of agriculture in the catchment on EPTr was not particularly relevant. Aggregation of different types of agriculture (e.g., intensive, extensive, organic, conventional, etc.) was probably confounding. Besides, impact is relative, and agriculture can be seen as positive for species richness compared with urbanization (Moore & Palmer, 2005) or negative when comparing it with forest (Fugère et al., 2016). The effect of hydrological predictors and slope used as proxy for flow velocity was relatively low. It is likely that these predictors did not account precisely enough for the actual processes controlling EPT responses (Poff & Ward, 1989; Ward, 1992). In addition, the relative influence of these processes may be weak when compared with other predictors related to pollution and thermal conditions, especially at such a large regional scale. Although the contribution of hydrological and temperature covariates was lower than land‐cover in the EPT species distribution and richness models, they played a significant role in the prediction phase when their values were modified under climate change scenarios.

Spatial eigenvectors used as covariates corresponded to large‐scale patterns and were relatively important for SDMs. They could be related to biological processes like species dispersal, and they probably enabled more accurate forecasts by constraining predictions of species’ range shift (Crase et al., 2014). In contrast, they had low influence on EPTr, which is likely to be controlled by more local factors.

Our results confirmed that species from higher elevation are more vulnerable to climate change (Besacier Monbertrand et al., 2019; Domisch et al., 2013). Indeed, under the RCP8.5 scenario for the 2080–2090 horizon, seven out of the 10 species most negatively impacted by climate change were sampled at a median elevation above 1,400 m a.s.l. Alpine species with low initial mean occurrence probability such as *Rhithrogena loyolaea*, *Protonemura brevistyla*, *Drusus discolor* or any rare alpine species whose distribution could not be modeled in this study are expected to be particularly vulnerable. In contrast, eight out of the 10 species most positively impacted by climate change were observed at a median elevation below 700 m a.s.l. However, considering all threats and not only climate change, the most endangered EPT species in Switzerland tend to be associated with large rivers (Lubini et al., 2012), usually related to low elevation and human activities. The highest increase in mean occurrence probability was for *Philopotamus ludificatus*, which was sampled in the montane zone, around 1,250 m a.s.l. This finding is in line with Timoner et al. (2020), who hypothesized that species from the montane level that are both eurythermic and rheophilic could be favored by global warming.

Median change of species occurrence probabilities was only slightly negative in the least conservative scenario (i.e., RCP8.5), but summing up all species, decrease in EPTr became noticeable. At the national scale, our results showed a reduction of around 8% with respect to the most conservative scenario. This is in accordance with the general relationship observed by Nunez et al. (2019) between climate change and biodiversity loss. However, some regions will likely to be more impacted than others. For instance, at the end of the century, decrease in EPTr in scenario RCP8.5 with respect to scenario RCP2.6 is expected to be of around 12% in Jura, while around only 3% in the Central Eastern Alps. Actually, higher regions (i.e., the Central Eastern Alps and Central Western Alps) seem less vulnerable in terms of EPTr, which partially supports findings from Brown et al. (2007). Their study showed that although climate change represents a risk for species adapted to alpine meltwater stream, new colonization will probably increase EPTr at high elevation. As increment in EPTr was mainly limited to very high elevation reaches, we found no evidence of an average increase in any of the Swiss biogeographic regions. Pattern of EPTr changes was also consistent with Li et al. (2013), who studied the same group of insects and found that higher elevation areas should be less vulnerable to global warming than lowlands, yet species loss at lower elevation in Switzerland could be compensated by new colonizations of warm‐adapted species from southern regions (Domisch et al., 2011, 2013). Overall, the relative decline in EPTr caused by climate change was much lower here than forecasted in other studies (~4% versus ~45% by mid‐century in scenario RCP8.5; Hamilton et al., 2010; Li et al., 2013). Finally, species may shift their realized niche within their fundamental niche in the future; thus, some of our projections may be misleading (Veloz et al., 2012). Although the spatial eigenvectors may have partially accounted for the large‐scale dispersal limitations, we did not take into account the dispersal modes and abilities of species (Årevall et al., 2018; Tonkin et al., 2018). Our modeling approach also ignored the biotic interactions (Post, 2013) and assumed a stable fundamental niche, neglecting any evolutionary response (De la Fuente et al., 2018). Although niche‐based models represent a useful tool for large‐scale conservation assessment (Araújo & Peterson, 2012), further research on metacommunity dynamics in the face of climate change are needed (e.g., Altermatt et al., 2008; Parain et al., 2019).

Our analysis showed that in the most conservative scenario, occurrence probability of species and EPTr will probably remain stable over time. Oppositely, in the least conservative scenario, decrease in EPTr is likely to be notable. While this latter scenario has been characterized as alarmist and deceitful (Hausfather & Peters, 2020), other researchers have argued that first, it is still highly plausible under current policies, and second, it serves to quantify climate risk (Schwalm et al., 2020). In any case, it is likely that in regions affected by high human influence, other factors like water pollution and degradation of habitats represent higher threats than climate change (Dudgeon et al., 2006; Reid et al., 2019). Therefore, any policy to conserve biodiversity in Switzerland should primarily focus on pollution reduction/limitation and habitat protection/restoration.

Predictions from MEM and S‐SDMs were congruent, which strengthened our findings. Our results confirmed that stacking raw occurrence probabilities, unlike binary predictions after thresholding them, do not lead to a systematic tendency to overpredict species richness, as commonly observed (Algar et al., 2009; Dubuis et al., 2011). Calabrese et al. (2014) found that overprediction in species‐poor sites and underprediction in species‐rich sites consistently occur independently of the chosen approach. In our study, S‐SDMs did not exhibit this pattern and performed slightly better than MEM. In any case, the low ratio of common and rare species probably reduced the prediction bias. SDM reliability has been shown to be lower for both common and rare species (McPherson et al., 2004; Santika, 2011). In Switzerland, environmental conditions are strongly related to elevation, and altitude preferences of species were similar for both the entire species pool and the selected species. We therefore considered our subset with no rare species as a fair proxy for EPTr assessment.

We integrated, for the first time to our knowledge, a coupled model for hydrology and glacier retreat into running water biodiversity models; however, not all the glaciers could be taken into account, each of them requiring a specific calibration. Furthermore, due to computational limitations, the SWAT model had to be calibrated at a monthly instead of a daily time‐step, which prevented us from using finer hydrological predictors related to flood characteristics. We consider that these limitations were a consequence of the methodological trade‐off between the spatial and temporal scales of our study and the resolution and precision of the environmental data. We are also aware that uncertainties in climatic and hydrological variables inevitably propagated along the modeling chain. As failing to account for them may have attenuated the variable responses (Stoklosa et al., 2015), climate change impacts on EPT may have been underestimated.

## 5 CONCLUSION

Although disentangling the effects of climate change, pollution and habitat degradation is difficult, we gave here an insight into the individual potential impacts of climate change on EPT species distribution and richness in running waters of a large temperate alpine region. Two complementary approaches consistently showed that overall, climate change is likely to reduce EPTr. Changes may be significant and decrease in species richness could be around 10% in the least conservative scenario, depending on the region. Overall, global warming was shown to represent a threat to species from high elevation, but in terms of EPTr, running waters from lowlands and medium elevation seemed more vulnerable. Furthermore, drivers of EPT species distribution and richness seem to be mostly related to land‐cover. Therefore, keeping in mind the importance of limiting climate change, we encourage policymakers from densely populated territory to give maximum attention to water pollution and habitat degradation for freshwater biodiversity conservation. The impact of climate change on freshwater systems will occur in the context of human activities, and biodiversity will probably be affected by the cumulative and potentially synergistic effects of multiple stressors.

## Supporting information

Supplementary Material

## Data Availability

Restrictions apply to the availability of these data, which were used under license for this study.
