# Supplementary material for: Impacts of climate change on aquatic insects in temperate alpine regions: Complementary modeling approaches applied to Swiss rivers
Source: Glob Chang Biol. 2021 May 20;27(15):3565–81. doi: 10.1111/gcb.15637 (PMC8360013; doi:10.1111/gcb.15637)
Supplement: Supplementary file 1 — Supplementary Material [file GCB-27-3565-s001.pdf]

Supplementary Material

Appendix 1

Catchments were divided into sub-catchments which included multiple hydrologic response units (HRUs). HRUs represented unique combinations of land-cover, soil and slope classes with associated land management practices. Calculation of water balance (Eq. 1) was made at the HRU level and results were aggregated at the sub-catchment scale.

$$SW_t = SW_0 + \sum_{i=1}^{t} \left(R_{day} - Q_{surf} - E_a - W_{seep} - Q_{gw} \right) \tag{1}$$

where SW_t = final soil water content (mm H_2O); SW_0 = initial soil water content (mm H_2O); t = time (days); R_{day} = amount of precipitation on day i (mm H_2O); Q_{surf} = amount of surface runoff on day i (mm H_2O); E_a = amount of evapotranspiration on day i (mm H_2O); W_{seep} = amount of water entering the vadose zone from the soil profile on day i (mm H_2O); Q_{gw} = amount of return flow on day i (mm H_2O).

Appendix 2

Percent bias (PBIAS) (Eq. 2) was measured during calibration for each gauging station. It measures the average tendency of the simulated data to be higher or lower than the observations. Optimum value is zero, where low magnitude values indicate better simulations. Positive values indicate model underestimation and negative values indicate model overestimation (Gupta *et al.*, 1999).

$$PBIAS = 100 \times \frac{\sum_{i=1}^{n} (Q_m - Q_s)_i}{\sum_{i=1}^{n} Q_{m,i}} \tag{2}$$

where Q is the discharge, and m and s stand for measured and simulated, respectively.

Appendix 3

Table S3.1: Fixed effects of the linear mixed model that was used to predict water temperature (AirTemp = air temperature; GlaCov = proportion of glacier cover in the catchment; LakeCov = proportion of lake cover in the catchment)

	**Estimate**	**Std. Error**	**df**	**t-value**	**Pr(>|t|)**
(Intercept)	3.47168	0.05002	2379.36951	69.407	< 0.0001
AirTemp	0.58523	0.01838	70.00089	31.834	< 0.0001
AirTemp:GlaCov	-1.26628	0.15134	68.08524	-8.367	< 0.0001
AirTemp:LakeCov	5.14921	0.76063	64.21966	6.770	< 0.0001

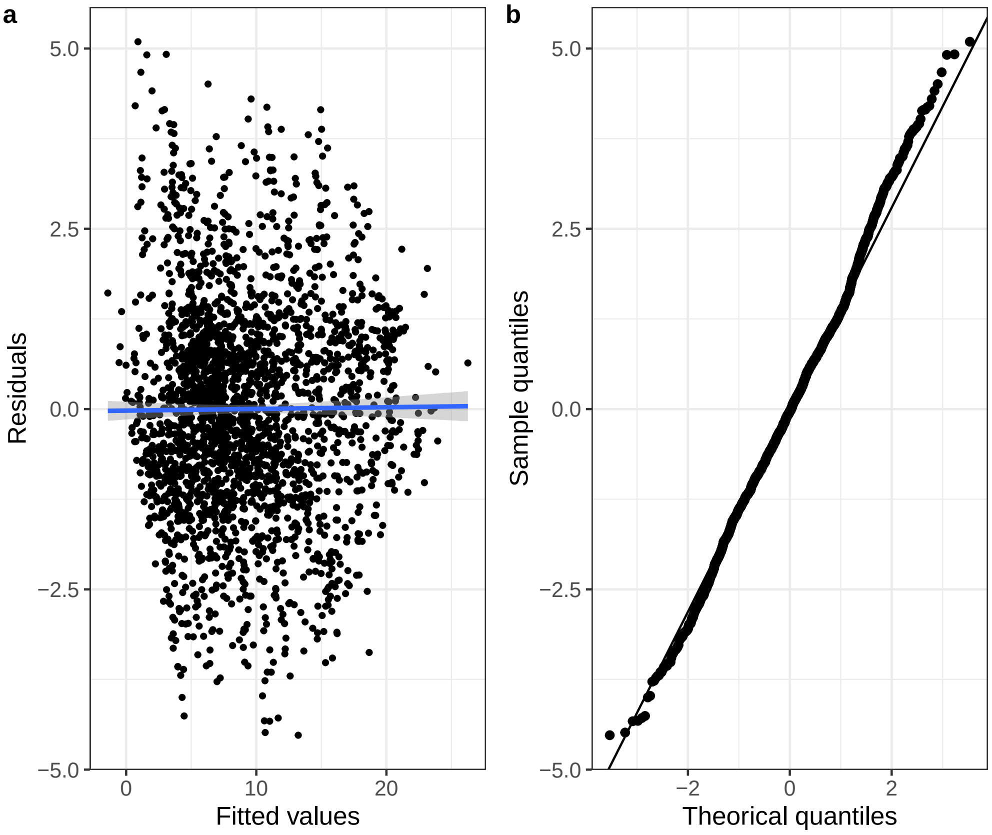

Figure S3.1: Diagnostic plots of the linear mixed model used to predict water temperature. Residuals versus fitted values (a) and quantile-quantile plot of residuals (b).

Appendix 4

Table S4.2: Climate model projections including GCM (General Circulation Model), RCM (Regional Climate Model) and resolution (12 km grid: EUR11 and 50 km grid: EUR44).

Projection name	RCM	GCM	Resolution
SMHI-RCA-ECEARTH-EUR11	RCA4	EC-EARTH	EUR11
MPICSC-REMO2-MPIESM-EUR44	REMO2009	MPI-ESM-LR	EUR44

Appendix 5

Table S5.3: Changes in mean occurrence probability ($\overline{p}$) of species according to scenario RCP2.6 in the Jura. Relative change (%) is calculated with respect to period 2015-2025. Species are ordered according to the relative change in 2080-2090.

Species	2015-2025	2055-2065		2080-2090	
	$\overline{p}$	$\overline{p}$	Δ (%)	$\overline{p}$	Δ (%)
Epeorus alpicola	0.0176	0.0137	-22.2%	0.0154	-12.7%
Protonemura nimborum	0.0178	0.0144	-19.0%	0.0162	-9.0%
Rhithrogena loyolaea	0.0033	0.0028	-15.4%	0.0031	-8.0%
Drusus discolor	0.0155	0.0130	-16.1%	0.0143	-7.9%
Chloroperla susemicheli	0.0374	0.0308	-17.5%	0.0347	-7.2%
Ecdyonurus picteti	0.0356	0.0285	-20.0%	0.0331	-7.0%
Rhithrogena alpestris	0.0271	0.0238	-12.2%	0.0256	-5.7%
Protonemura brevistyla	0.0024	0.0021	-11.3%	0.0023	-5.6%
Nemoura mortoni	0.1065	0.0984	-7.6%	0.1017	-4.5%
Rhithrogena puthzi	0.0249	0.0225	-9.4%	0.0240	-3.7%
Ecdyonurus helveticus	0.1647	0.1584	-3.8%	0.1593	-3.3%
Protonemura lateralis	0.0661	0.0634	-4.1%	0.0645	-2.5%
Philopotamus ludificatus	0.0266	0.0272	+2.5%	0.0261	-1.9%
Rhyacophila tristis	0.6494	0.6325	-2.6%	0.6373	-1.9%
Baetis alpinus	0.5401	0.5240	-3.0%	0.5314	-1.6%
Nemoura minima	0.2293	0.2211	-3.6%	0.2258	-1.5%
Habrophlebia lauta	0.2521	0.2414	-4.2%	0.2495	-1.0%
Halesus digitatus	0.4295	0.4221	-1.7%	0.4260	-0.8%
Potamophylax cingulatus	0.5102	0.5056	-0.9%	0.5060	-0.8%
Serratella ignita	0.2816	0.2858	+1.5%	0.2793	-0.8%
Brachyptera risi	0.5039	0.4977	-1.2%	0.5003	-0.7%
Isoperla rivulorum	0.0323	0.0318	-1.4%	0.0321	-0.6%
Amphinemura sulcicollis	0.3515	0.3478	-1.0%	0.3495	-0.5%
Odontocerum albicorne	0.4729	0.4695	-0.7%	0.4706	-0.5%
Baetis muticus	0.6215	0.6165	-0.8%	0.6188	-0.4%
Capnioneura nemuroides	0.1902	0.1853	-2.5%	0.1894	-0.4%
Epeorus assimilis	0.4195	0.4194	-0.0%	0.4184	-0.3%
Allogamus auricollis	0.5195	0.5128	-1.3%	0.5188	-0.1%
Baetis rhodani	0.9798	0.9812	+0.1%	0.9802	0.0%
Centroptilum luteolum	0.3268	0.3225	-1.3%	0.3268	-0.0%
Ecdyonurus torrentis	0.1079	0.1048	-2.8%	0.1079	0.0%
Rhyacophila torrentium	0.0360	0.0349	-3.2%	0.0360	0.0%
Habroleptoides confusa	0.4801	0.4787	-0.3%	0.4807	+0.1%
Rhithrogena semicolorata	0.5472	0.5465	-0.1%	0.5479	+0.1%
Ecdyonurus venosus	0.3936	0.3929	-0.2%	0.3944	+0.2%
Isoperla grammatica	0.2119	0.2129	+0.4%	0.2123	+0.2%
Paraleptophlebia submarginata	0.2471	0.2471	0.0%	0.2475	+0.2%
Perla grandis	0.1126	0.1111	-1.3%	0.1128	+0.2%
Baetis lutheri	0.5629	0.5664	+0.6%	0.5676	+0.8%
Hydropsyche siltalai	0.2902	0.3005	+3.5%	0.2951	+1.7%
Ephemera danica	0.2003	0.2072	+3.4%	0.2045	+2.1%

Table S5.4: Changes in mean occurrence probability ($\overline{p}$) of species according to scenario RCP8.5 in the Jura. Relative change (%) is calculated with respect to period 2015-2025. Species are ordered according to the relative change in 2080-2090.

**Species**	**2015-2025**	**2055-2065**		**2080-2090**	
	$\overline{p}$	$\overline{p}$	Δ (%)	$\overline{p}$	Δ (%)
Protonemura nimborum	0.0184	0.0088	-52.3%	0.0079	-56.9%
Nemoura mortoni	0.1087	0.0699	-35.8%	0.0556	-48.9%
Ecdyonurus picteti	0.0349	0.0194	-44.4%	0.0179	-48.7%
Rhithrogena alpestris	0.0272	0.0167	-38.7%	0.0148	-45.7%
Rhithrogena puthzi	0.0251	0.0171	-31.9%	0.0151	-39.9%
Chloroperla susemicheli	0.0372	0.0230	-38.2%	0.0230	-38.1%
Epeorus alpicola	0.0179	0.0115	-35.6%	0.0116	-35.1%
Drusus discolor	0.0157	0.0103	-34.8%	0.0105	-33.6%
Nemoura minima	0.2285	0.1796	-21.4%	0.1553	-32.0%
Baetis alpinus	0.5476	0.4556	-16.8%	0.3760	-31.3%
Rhyacophila torrentium	0.0363	0.0276	-24.0%	0.0251	-30.8%
Ecdyonurus helveticus	0.1682	0.1338	-20.5%	0.1219	-27.5%
Rhyacophila tristis	0.6586	0.5654	-14.2%	0.4835	-26.6%
Rhithrogena loyolaea	0.0034	0.0024	-30.4%	0.0025	-26.3%
Capnioneura nemuroides	0.1882	0.1589	-15.6%	0.1401	-25.6%
Protonemura lateralis	0.0670	0.0539	-19.5%	0.0502	-25.2%
Brachyptera risi	0.5063	0.4660	-8.0%	0.4078	-19.5%
Perla grandis	0.1133	0.0963	-15.0%	0.0915	-19.2%
Habrophlebia lauta	0.2439	0.2108	-13.6%	0.2019	-17.2%
Ecdyonurus venosus	0.3996	0.3676	-8.0%	0.3317	-17.0%
Amphinemura sulcicollis	0.3525	0.3212	-8.9%	0.2984	-15.4%
Halesus digitatus	0.4285	0.3953	-7.8%	0.3715	-13.3%
Protonemura brevistyla	0.0024	0.0019	-18.7%	0.0021	-13.1%
Isoperla grammatica	0.2140	0.2018	-5.7%	0.1917	-10.5%
Potamophylax cingulatus	0.5137	0.4871	-5.2%	0.4632	-9.8%
Rhithrogena semicolorata	0.5461	0.5384	-1.4%	0.4964	-9.1%
Allogamus auricollis	0.5147	0.4878	-5.2%	0.4717	-8.4%
Baetis lutheri	0.5639	0.5642	+0.1%	0.5167	-8.4%
Odontocerum albicorne	0.4702	0.4537	-3.5%	0.4410	-6.2%
Baetis muticus	0.6181	0.6031	-2.4%	0.5830	-5.7%
Ecdyonurus torrentis	0.1059	0.1011	-4.5%	0.1031	-2.6%
Serratella ignita	0.2928	0.2988	+2.0%	0.2887	-1.4%
Baetis rhodani	0.9803	0.9842	+0.4%	0.9835	+0.3%
Epeorus assimilis	0.4235	0.4243	+0.2%	0.4246	+0.3%
Centroptilum luteolum	0.3226	0.3150	-2.3%	0.3258	+1.0%
Habroleptoides confusa	0.4767	0.4774	+0.1%	0.4924	+3.3%
Isoperla rivulorum	0.0329	0.0288	-12.5%	0.0344	+4.5%
Hydropsyche siltalai	0.2966	0.3356	+13.2%	0.3607	+21.6%
Paraleptophlebia submarginata	0.2421	0.2662	+9.9%	0.2965	+22.4%
Ephemera danica	0.1959	0.2358	+20.3%	0.3095	+58.0%
Philopotamus ludificatus	0.0269	0.0403	+49.8%	0.0580	+115.8%

Table S5.5: Changes in mean occurrence probability ($\overline{p}$) of species according to scenario RCP2.6 in the Plateau. Relative change (%) is calculated with respect to period 2015-2025. Species are ordered according to the relative change in 2080-2090.

**Species**	**2015-2025**	**2055-2065**		**2080-2090**	
	$\overline{p}$	$\overline{p}$	Δ (%)	$\overline{p}$	Δ (%)
Nemoura minima	0.0961	0.0919	-4.3%	0.0915	-4.8%
Protonemura nimborum	0.0078	0.0074	-5.3%	0.0074	-4.6%
Capnioneura nemuroides	0.0597	0.0570	-4.5%	0.0572	-4.1%
Nemoura mortoni	0.0263	0.0252	-4.3%	0.0252	-4.1%
Rhyacophila tristis	0.4294	0.4109	-4.3%	0.4118	-4.1%
Protonemura lateralis	0.0269	0.0266	-1.2%	0.0259	-3.9%
Baetis alpinus	0.2742	0.2594	-5.4%	0.2653	-3.2%
Chloroperla susemicheli	0.0059	0.0058	-2.1%	0.0057	-3.2%
Ecdyonurus helveticus	0.0924	0.0902	-2.3%	0.0894	-3.2%
Epeorus alpicola	0.0042	0.0042	-1.5%	0.0041	-3.2%
Serratella ignita	0.2356	0.2416	+2.5%	0.2284	-3.1%
Drusus discolor	0.0051	0.0051	-1.3%	0.0050	-3.0%
Rhithrogena puthzi	0.0161	0.0155	-3.8%	0.0157	-2.7%
Amphinemura sulcicollis	0.1818	0.1789	-1.6%	0.1777	-2.3%
Brachyptera risi	0.3603	0.3521	-2.3%	0.3523	-2.2%
Allogamus auricollis	0.3542	0.3460	-2.3%	0.3475	-1.9%
Potamophylax cingulatus	0.3539	0.3502	-1.1%	0.3475	-1.8%
Habrophlebia lauta	0.2519	0.2352	-6.6%	0.2480	-1.6%
Isoperla grammatica	0.2101	0.2080	-1.0%	0.2072	-1.4%
Odontocerum albicorne	0.2965	0.2925	-1.3%	0.2925	-1.4%
Perla grandis	0.0457	0.0451	-1.2%	0.0450	-1.4%
Ecdyonurus picteti	0.0043	0.0042	-1.2%	0.0042	-1.3%
Isoperla rivulorum	0.0299	0.0297	-0.7%	0.0296	-1.3%
Baetis lutheri	0.4779	0.4738	-0.9%	0.4722	-1.2%
Halesus digitatus	0.4153	0.4051	-2.4%	0.4106	-1.1%
Rhithrogena semicolorata	0.3947	0.3897	-1.3%	0.3904	-1.1%
Rhithrogena alpestris	0.0085	0.0083	-1.7%	0.0084	-0.8%
Rhyacophila torrentium	0.0367	0.0360	-1.9%	0.0364	-0.8%
Centroptilum luteolum	0.2535	0.2492	-1.7%	0.2518	-0.7%
Baetis muticus	0.4209	0.4156	-1.3%	0.4183	-0.6%
Ecdyonurus venosus	0.3283	0.3240	-1.3%	0.3264	-0.6%
Rhithrogena loyolaea	0.0034	0.0034	+0.6%	0.0034	-0.4%
Epeorus assimilis	0.1914	0.1910	-0.2%	0.1912	-0.1%
Baetis rhodani	0.9547	0.9559	+0.1%	0.9555	+0.1%
Paraleptophlebia submarginata	0.2881	0.2833	-1.7%	0.2892	+0.4%
Habroleptoides confusa	0.3049	0.2992	-1.9%	0.3063	+0.5%
Hydropsyche siltalai	0.4075	0.4141	+1.6%	0.4103	+0.7%
Protonemura brevistyla	0.0034	0.0034	+0.8%	0.0034	+0.8%
Philopotamus ludificatus	0.0125	0.0136	+8.8%	0.0127	+1.8%
Ecdyonurus torrentis	0.1401	0.1354	-3.3%	0.1437	+2.6%
Ephemera danica	0.2283	0.2358	+3.3%	0.2361	+3.4%

Table S5.6: Changes in mean occurrence probability ($\overline{p}$) of species according to scenario RCP8.5 in the Plateau. Relative change (%) is calculated with respect to period 2015-2025. Species are ordered according to the relative change in 2080-2090.

**Species**	**2015-2025**	**2055-2065**		**2080-2090**	
	$\overline{p}$	$\overline{p}$	Δ (%)	$\overline{p}$	Δ (%)
Baetis alpinus	0.2771	0.2159	-22.1%	0.1864	-32.7%
Rhyacophila tristis	0.4327	0.3514	-18.8%	0.3028	-30.0%
Nemoura mortoni	0.0271	0.0229	-15.4%	0.0200	-26.1%
Habrophlebia lauta	0.2459	0.1880	-23.5%	0.1839	-25.2%
Capnioneura nemuroides	0.0592	0.0533	-9.9%	0.0478	-19.2%
Brachyptera risi	0.3613	0.3310	-8.4%	0.2934	-18.8%
Rhithrogena puthzi	0.0163	0.0140	-14.3%	0.0135	-17.4%
Nemoura minima	0.0953	0.0880	-7.6%	0.0799	-16.2%
Ecdyonurus venosus	0.3315	0.3091	-6.7%	0.2869	-13.5%
Rhithrogena alpestris	0.0086	0.0078	-9.2%	0.0075	-13.2%
Halesus digitatus	0.4127	0.3761	-8.9%	0.3600	-12.8%
Chloroperla susemicheli	0.0060	0.0055	-7.9%	0.0052	-12.1%
Rhithrogena semicolorata	0.3945	0.3748	-5.0%	0.3477	-11.9%
Protonemura nimborum	0.0080	0.0068	-14.5%	0.0070	-11.7%
Baetis lutheri	0.4783	0.4678	-2.2%	0.4314	-9.8%
Ecdyonurus picteti	0.0043	0.0040	-7.2%	0.0039	-9.3%
Rhyacophila torrentium	0.0368	0.0345	-6.3%	0.0334	-9.3%
Amphinemura sulcicollis	0.1821	0.1792	-1.6%	0.1656	-9.0%
Odontocerum albicorne	0.2931	0.2854	-2.6%	0.2697	-8.0%
Ecdyonurus torrentis	0.1371	0.1204	-12.2%	0.1275	-7.0%
Isoperla grammatica	0.2112	0.2040	-3.4%	0.1999	-5.4%
Potamophylax cingulatus	0.3548	0.3478	-2.0%	0.3407	-4.0%
Allogamus auricollis	0.3493	0.3370	-3.5%	0.3359	-3.8%
Ecdyonurus helveticus	0.0936	0.0909	-2.9%	0.0906	-3.3%
Baetis muticus	0.4182	0.4092	-2.1%	0.4057	-3.0%
Perla grandis	0.0467	0.0452	-3.3%	0.0461	-1.4%
Centroptilum luteolum	0.2492	0.2366	-5.0%	0.2472	-0.8%
Protonemura lateralis	0.0275	0.0282	+2.7%	0.0275	+0.1%
Baetis rhodani	0.9556	0.9591	+0.4%	0.9575	+0.2%
Habroleptoides confusa	0.3026	0.2887	-4.6%	0.3043	+0.6%
Protonemura brevistyla	0.0034	0.0034	+1.0%	0.0035	+3.3%
Rhithrogena loyolaea	0.0034	0.0033	-0.7%	0.0035	+4.3%
Drusus discolor	0.0051	0.0052	+2.8%	0.0053	+4.5%
Paraleptophlebia submarginata	0.2836	0.2792	-1.6%	0.3080	+8.6%
Epeorus assimilis	0.1931	0.1973	+2.2%	0.2160	+11.8%
Hydropsyche siltalai	0.4163	0.4409	+5.9%	0.4673	+12.2%
Serratella ignita	0.2413	0.2810	+16.5%	0.2833	+17.4%
Isoperla rivulorum	0.0301	0.0289	-4.2%	0.0357	+18.5%
Epeorus alpicola	0.0043	0.0048	+10.0%	0.0052	+20.0%
Ephemera danica	0.2246	0.2618	+16.6%	0.3220	+43.4%
Philopotamus ludificatus	0.0127	0.0214	+69.2%	0.0288	+127.7%

Table S5.7: Changes in mean occurrence probability ($\overline{p}$) of species according to scenario RCP2.6 in the Northern Alps. Relative change (%) is calculated with respect to period 2015-2025. Species are ordered according to the relative change in 2080-2090.

**Species**	**2015-2025**	**2055-2065**		**2080-2090**	
	$\overline{p}$	$\overline{p}$	Δ (%)	$\overline{p}$	Δ (%)
Rhithrogena loyolaea	0.0786	0.0637	-19.0%	0.0647	-17.7%
Protonemura brevistyla	0.0757	0.0658	-13.1%	0.0665	-12.2%
Serratella ignita	0.0954	0.0877	-8.0%	0.0850	-10.9%
Nemoura minima	0.3483	0.3232	-7.2%	0.3294	-5.4%
Capnioneura nemuroides	0.3039	0.2860	-5.9%	0.2896	-4.7%
Protonemura nimborum	0.1189	0.1119	-5.9%	0.1150	-3.3%
Rhithrogena puthzi	0.2290	0.2178	-4.9%	0.2225	-2.8%
Baetis lutheri	0.1832	0.1807	-1.4%	0.1782	-2.7%
Allogamus auricollis	0.5516	0.5366	-2.7%	0.5385	-2.4%
Perla grandis	0.2311	0.2239	-3.1%	0.2263	-2.1%
Ecdyonurus picteti	0.1965	0.1872	-4.7%	0.1925	-2.0%
Rhyacophila tristis	0.4638	0.4483	-3.4%	0.4548	-2.0%
Rhithrogena alpestris	0.1955	0.1874	-4.2%	0.1927	-1.4%
Halesus digitatus	0.2361	0.2310	-2.2%	0.2330	-1.3%
Odontocerum albicorne	0.1888	0.1863	-1.3%	0.1863	-1.3%
Brachyptera risi	0.3823	0.3770	-1.4%	0.3776	-1.2%
Isoperla grammatica	0.2505	0.2477	-1.1%	0.2474	-1.2%
Nemoura mortoni	0.3896	0.3796	-2.6%	0.3858	-1.0%
Ecdyonurus venosus	0.2866	0.2824	-1.5%	0.2841	-0.9%
Amphinemura sulcicollis	0.4401	0.4364	-0.8%	0.4372	-0.7%
Rhyacophila torrentium	0.2449	0.2398	-2.1%	0.2431	-0.7%
Baetis alpinus	0.8507	0.8418	-1.0%	0.8464	-0.5%
Potamophylax cingulatus	0.3361	0.3362	0.0%	0.3349	-0.3%
Rhithrogena semicolorata	0.4162	0.4157	-0.1%	0.4151	-0.2%
Chloroperla susemicheli	0.2159	0.2117	-1.9%	0.2158	-0.0%
Habrophlebia lauta	0.0925	0.0905	-2.1%	0.0926	+0.2%
Ecdyonurus helveticus	0.5091	0.5064	-0.5%	0.5111	+0.4%
Drusus discolor	0.1233	0.1196	-3.0%	0.1238	+0.5%
Baetis muticus	0.5150	0.5198	+0.9%	0.5181	+0.6%
Isoperla rivulorum	0.1277	0.1273	-0.3%	0.1290	+1.0%
Protonemura lateralis	0.3423	0.3431	+0.2%	0.3461	+1.1%
Habroleptoides confusa	0.2656	0.2682	+1.0%	0.2689	+1.2%
Centroptilum luteolum	0.0909	0.0924	+1.7%	0.0921	+1.3%
Epeorus assimilis	0.3095	0.3155	+1.9%	0.3139	+1.4%
Baetis rhodani	0.8494	0.8685	+2.2%	0.8657	+1.9%
Hydropsyche siltalai	0.1004	0.1054	+5.0%	0.1027	+2.3%
Epeorus alpicola	0.1653	0.1666	+0.8%	0.1705	+3.1%
Paraleptophlebia submarginata	0.1133	0.1201	+6.0%	0.1187	+4.7%
Ephemera danica	0.0756	0.0810	+7.2%	0.0792	+4.8%
Ecdyonurus torrentis	0.0466	0.0483	+3.7%	0.0492	+5.5%
Philopotamus ludificatus	0.0973	0.1096	+12.6%	0.1075	+10.5%

Table S5.8: Changes in mean occurrence probability ($\overline{p}$) of species according to scenario RCP8.5 in the Northern Alps. Relative change (%) is calculated with respect to period 2015-2025. Species are ordered according to the relative change in 2080-2090.

**Species**	**2015-2025**	**2055-2065**		**2080-2090**	
	$\overline{p}$	$\overline{p}$	Δ (%)	$\overline{p}$	Δ (%)
Rhithrogena loyolaea	0.0758	0.0449	-40.8%	0.0294	-61.2%
Protonemura brevistyla	0.0738	0.0511	-30.7%	0.0382	-48.2%
Rhithrogena alpestris	0.1926	0.1527	-20.7%	0.1240	-35.6%
Protonemura nimborum	0.1151	0.0931	-19.1%	0.0767	-33.4%
Ecdyonurus picteti	0.1919	0.1547	-19.4%	0.1309	-31.8%
Rhithrogena puthzi	0.2252	0.1857	-17.5%	0.1547	-31.3%
Rhyacophila tristis	0.4578	0.4062	-11.3%	0.3265	-28.7%
Drusus discolor	0.1195	0.1027	-14.0%	0.0871	-27.1%
Nemoura mortoni	0.3832	0.3404	-11.2%	0.2942	-23.2%
Rhyacophila torrentium	0.2406	0.2142	-11.0%	0.1870	-22.3%
Nemoura minima	0.3311	0.2972	-10.3%	0.2650	-20.0%
Habrophlebia lauta	0.0920	0.0781	-15.2%	0.0753	-18.2%
Baetis alpinus	0.8510	0.7899	-7.2%	0.7125	-16.3%
Ecdyonurus helveticus	0.5092	0.4771	-6.3%	0.4415	-13.3%
Ecdyonurus venosus	0.2862	0.2718	-5.0%	0.2512	-12.2%
Halesus digitatus	0.2334	0.2175	-6.8%	0.2067	-11.4%
Capnioneura nemuroides	0.2873	0.2753	-4.2%	0.2549	-11.3%
Epeorus alpicola	0.1637	0.1568	-4.2%	0.1483	-9.4%
Chloroperla susemicheli	0.2116	0.1993	-5.8%	0.1927	-8.9%
Allogamus auricollis	0.5420	0.5110	-5.7%	0.4974	-8.2%
Brachyptera risi	0.3766	0.3810	+1.2%	0.3467	-7.9%
Ecdyonurus torrentis	0.0482	0.0436	-9.5%	0.0453	-6.0%
Isoperla grammatica	0.2497	0.2467	-1.2%	0.2389	-4.3%
Amphinemura sulcicollis	0.4367	0.4359	-0.2%	0.4199	-3.8%
Protonemura lateralis	0.3394	0.3403	+0.3%	0.3304	-2.7%
Rhithrogena semicolorata	0.4120	0.4261	+3.4%	0.4046	-1.8%
Isoperla rivulorum	0.1253	0.1247	-0.5%	0.1234	-1.6%
Perla grandis	0.2248	0.2215	-1.5%	0.2216	-1.4%
Habroleptoides confusa	0.2692	0.2630	-2.3%	0.2675	-0.6%
Potamophylax cingulatus	0.3339	0.3449	+3.3%	0.3383	+1.3%
Odontocerum albicorne	0.1870	0.1882	+0.6%	0.1927	+3.0%
Baetis muticus	0.5139	0.5382	+4.7%	0.5411	+5.3%
Epeorus assimilis	0.3155	0.3213	+1.8%	0.3354	+6.3%
Baetis rhodani	0.8546	0.8995	+5.3%	0.9237	+8.1%
Baetis lutheri	0.1759	0.1972	+12.1%	0.1926	+9.5%
Centroptilum luteolum	0.0896	0.0984	+9.8%	0.1121	+25.1%
Paraleptophlebia submarginata	0.1159	0.1305	+12.6%	0.1498	+29.2%
Hydropsyche siltalai	0.1028	0.1275	+24.0%	0.1539	+49.6%
Serratella ignita	0.0879	0.1187	+35.0%	0.1386	+57.6%
Ephemera danica	0.0760	0.1000	+31.5%	0.1339	+76.1%
Philopotamus ludificatus	0.1021	0.1530	+49.8%	0.2177	+113.1%

Table S5.9: Changes in mean occurrence probability ($\overline{p}$) of species according to scenario RCP2.6 in the Central Eastern Alps. Relative change (%) is calculated with respect to period 2015-2025. Species are ordered according to the relative change in 2080-2090.

**Species**	**2015-2025**	**2055-2065**		**2080-2090**	
	$\overline{p}$	$\overline{p}$	Δ (%)	$\overline{p}$	Δ (%)
Habrophlebia lauta	0.0229	0.0211	-7.9%	0.0217	-5.5%
Rhithrogena loyolaea	0.2445	0.2227	-8.9%	0.2322	-5.0%
Nemoura minima	0.1794	0.1736	-3.2%	0.1746	-2.7%
Capnioneura nemuroides	0.0907	0.0886	-2.3%	0.0885	-2.4%
Halesus digitatus	0.1081	0.1019	-5.7%	0.1057	-2.2%
Protonemura brevistyla	0.2710	0.2630	-2.9%	0.2649	-2.2%
Odontocerum albicorne	0.0559	0.0542	-3.0%	0.0549	-1.7%
Centroptilum luteolum	0.0211	0.0212	0.0%	0.0209	-1.3%
Rhithrogena puthzi	0.5727	0.5562	-2.9%	0.5671	-1.0%
Ecdyonurus torrentis	0.0123	0.0120	-1.9%	0.0122	-0.9%
Rhyacophila tristis	0.3983	0.3860	-3.1%	0.3956	-0.7%
Brachyptera risi	0.1378	0.1370	-0.6%	0.1371	-0.5%
Drusus discolor	0.3349	0.3257	-2.8%	0.3332	-0.5%
Allogamus auricollis	0.5078	0.5123	+0.9%	0.5058	-0.4%
Habroleptoides confusa	0.0919	0.0911	-0.8%	0.0917	-0.2%
Serratella ignita	0.0207	0.0201	-3.0%	0.0206	-0.2%
Baetis muticus	0.2878	0.2881	+0.1%	0.2878	0.0%
Baetis alpinus	0.9713	0.9696	-0.2%	0.9711	-0.0%
Epeorus alpicola	0.3816	0.3731	-2.2%	0.3822	+0.1%
Nemoura mortoni	0.6753	0.6717	-0.5%	0.6763	+0.1%
Chloroperla susemicheli	0.2661	0.2675	+0.5%	0.2668	+0.3%
Baetis lutheri	0.0326	0.0331	+1.5%	0.0328	+0.4%
Protonemura nimborum	0.1706	0.1715	+0.6%	0.1712	+0.4%
Rhithrogena semicolorata	0.1898	0.1930	+1.7%	0.1905	+0.4%
Perla grandis	0.2000	0.2016	+0.8%	0.2014	+0.7%
Potamophylax cingulatus	0.1252	0.1287	+2.8%	0.1261	+0.7%
Protonemura lateralis	0.5802	0.5811	+0.2%	0.5842	+0.7%
Rhithrogena alpestris	0.4791	0.4804	+0.3%	0.4826	+0.7%
Amphinemura sulcicollis	0.3042	0.3082	+1.3%	0.3067	+0.8%
Ecdyonurus helveticus	0.6378	0.6410	+0.5%	0.6428	+0.8%
Ecdyonurus picteti	0.3732	0.3764	+0.9%	0.3767	+0.9%
Isoperla grammatica	0.0969	0.0986	+1.8%	0.0979	+1.1%
Ecdyonurus venosus	0.1346	0.1350	+0.3%	0.1365	+1.4%
Baetis rhodani	0.6157	0.6291	+2.2%	0.6249	+1.5%
Epeorus assimilis	0.1475	0.1508	+2.2%	0.1498	+1.5%
Hydropsyche siltalai	0.0175	0.0184	+4.9%	0.0179	+2.0%
Philopotamus ludificatus	0.1614	0.1629	+0.9%	0.1656	+2.6%
Rhyacophila torrentium	0.3730	0.3900	+4.6%	0.3837	+2.9%
Paraleptophlebia submarginata	0.0258	0.0276	+7.0%	0.0267	+3.3%
Isoperla rivulorum	0.1830	0.1922	+5.1%	0.1900	+3.9%
Ephemera danica	0.0145	0.0161	+11.1%	0.0152	+4.9%

Table S5.10: Changes in mean occurrence probability ($\overline{p}$) of species according to scenario RCP8.5 in the Central Eastern Alps. Relative change (%) is calculated with respect to period 2015-2025. Species are ordered according to the relative change in 2080-2090.

**Species**	**2015-2025**	**2055-2065**		**2080-2090**	
	$\overline{p}$	$\overline{p}$	Δ (%)	$\overline{p}$	Δ (%)
Rhithrogena loyolaea	0.2365	0.1699	-28.2%	0.1084	-54.1%
Protonemura brevistyla	0.2683	0.2204	-17.8%	0.1640	-38.9%
Rhithrogena puthzi	0.5674	0.4901	-13.6%	0.3968	-30.1%
Drusus discolor	0.3325	0.2837	-14.7%	0.2348	-29.4%
Rhithrogena alpestris	0.4814	0.4251	-11.7%	0.3549	-26.3%
Rhyacophila tristis	0.3953	0.3451	-12.7%	0.3021	-23.6%
Protonemura nimborum	0.1716	0.1543	-10.1%	0.1342	-21.8%
Ecdyonurus picteti	0.3744	0.3438	-8.2%	0.2933	-21.7%
Rhyacophila torrentium	0.3805	0.3555	-6.6%	0.3201	-15.9%
Epeorus alpicola	0.3791	0.3466	-8.6%	0.3199	-15.6%
Nemoura mortoni	0.6745	0.6453	-4.3%	0.5958	-11.7%
Halesus digitatus	0.1051	0.0969	-7.8%	0.0932	-11.3%
Isoperla rivulorum	0.1868	0.1773	-5.1%	0.1718	-8.0%
Ecdyonurus venosus	0.1350	0.1265	-6.2%	0.1243	-7.9%
Habrophlebia lauta	0.0225	0.0198	-12.0%	0.0208	-7.9%
Baetis alpinus	0.9708	0.9570	-1.4%	0.9221	-5.0%
Ecdyonurus helveticus	0.6406	0.6320	-1.3%	0.6159	-3.9%
Ecdyonurus torrentis	0.0124	0.0113	-8.9%	0.0123	-1.0%
Protonemura lateralis	0.5808	0.5848	+0.7%	0.5784	-0.4%
Nemoura minima	0.1743	0.1748	+0.3%	0.1762	+1.1%
Allogamus auricollis	0.5094	0.5183	+1.8%	0.5162	+1.3%
Chloroperla susemicheli	0.2670	0.2727	+2.1%	0.2736	+2.5%
Brachyptera risi	0.1361	0.1405	+3.2%	0.1436	+5.5%
Odontocerum albicorne	0.0559	0.0551	-1.5%	0.0590	+5.5%
Habroleptoides confusa	0.0920	0.0933	+1.4%	0.1003	+9.1%
Perla grandis	0.1973	0.2044	+3.6%	0.2189	+11.0%
Amphinemura sulcicollis	0.3060	0.3195	+4.4%	0.3412	+11.5%
Isoperla grammatica	0.0968	0.1031	+6.5%	0.1080	+11.6%
Rhithrogena semicolorata	0.1886	0.2128	+12.8%	0.2350	+24.6%
Baetis muticus	0.2867	0.3189	+11.2%	0.3630	+26.6%
Baetis rhodani	0.6171	0.7018	+13.7%	0.7840	+27.0%
Epeorus assimilis	0.1492	0.1679	+12.6%	0.1922	+28.8%
Capnioneura nemuroides	0.0854	0.0991	+16.1%	0.1138	+33.3%
Potamophylax cingulatus	0.1257	0.1523	+21.1%	0.1809	+43.8%
Baetis lutheri	0.0319	0.0388	+21.6%	0.0472	+47.9%
Philopotamus ludificatus	0.1642	0.1976	+20.4%	0.2657	+61.9%
Serratella ignita	0.0205	0.0215	+4.8%	0.0333	+62.6%
Paraleptophlebia submarginata	0.0267	0.0337	+26.1%	0.0446	+66.7%
Centroptilum luteolum	0.0206	0.0261	+26.5%	0.0345	+67.0%
Ephemera danica	0.0151	0.0201	+33.3%	0.0297	+97.3%
Hydropsyche siltalai	0.0180	0.0242	+34.6%	0.0370	+105.6%

Table S5.11: Changes in mean occurrence probability ($\overline{p}$) of species according to scenario RCP2.6 in the Southern Alps. Relative change (%) is calculated with respect to period 2015-2025. Species are ordered according to the relative change in 2080-2090.

**Species**	**2015-2025**	**2055-2065**		**2080-2090**	
	$\overline{p}$	$\overline{p}$	Δ (%)	$\overline{p}$	Δ (%)
Habrophlebia lauta	0.0246	0.0234	-4.9%	0.0236	-3.9%
Nemoura minima	0.1358	0.1256	-7.5%	0.1305	-3.9%
Rhithrogena loyolaea	0.1175	0.1101	-6.3%	0.1138	-3.1%
Brachyptera risi	0.1722	0.1645	-4.5%	0.1679	-2.5%
Capnioneura nemuroides	0.0690	0.0651	-5.7%	0.0674	-2.3%
Halesus digitatus	0.0621	0.0594	-4.4%	0.0606	-2.3%
Baetis muticus	0.3986	0.3890	-2.4%	0.3905	-2.0%
Drusus discolor	0.1718	0.1651	-3.9%	0.1692	-1.5%
Rhyacophila tristis	0.4165	0.4041	-3.0%	0.4101	-1.5%
Chloroperla susemicheli	0.1821	0.1775	-2.6%	0.1797	-1.4%
Potamophylax cingulatus	0.2749	0.2702	-1.7%	0.2710	-1.4%
Odontocerum albicorne	0.1997	0.1964	-1.6%	0.1971	-1.3%
Epeorus alpicola	0.2058	0.1991	-3.3%	0.2034	-1.2%
Habroleptoides confusa	0.1368	0.1356	-0.8%	0.1354	-1.0%
Nemoura mortoni	0.5004	0.4875	-2.6%	0.4953	-1.0%
Centroptilum luteolum	0.0309	0.0305	-1.2%	0.0306	-0.9%
Amphinemura sulcicollis	0.3050	0.3012	-1.2%	0.3029	-0.7%
Protonemura lateralis	0.4779	0.4716	-1.3%	0.4744	-0.7%
Paraleptophlebia submarginata	0.0514	0.0517	+0.5%	0.0513	-0.4%
Allogamus auricollis	0.4676	0.4636	-0.9%	0.4660	-0.3%
Rhithrogena puthzi	0.2409	0.2357	-2.1%	0.2402	-0.3%
Serratella ignita	0.1056	0.1067	+1.0%	0.1053	-0.3%
Baetis alpinus	0.9158	0.9135	-0.3%	0.9149	-0.1%
Ecdyonurus helveticus	0.6531	0.6503	-0.4%	0.6522	-0.1%
Rhithrogena semicolorata	0.4368	0.4371	+0.1%	0.4369	0.0%
Baetis rhodani	0.8422	0.8463	+0.5%	0.8428	+0.1%
Ecdyonurus torrentis	0.0129	0.0132	+2.5%	0.0129	+0.3%
Protonemura nimborum	0.1028	0.1014	-1.3%	0.1030	+0.3%
Baetis lutheri	0.0431	0.0429	-0.6%	0.0433	+0.5%
Protonemura brevistyla	0.1096	0.1086	-1.0%	0.1102	+0.5%
Epeorus assimilis	0.3521	0.3579	+1.7%	0.3544	+0.6%
Philopotamus ludificatus	0.2823	0.2905	+2.9%	0.2840	+0.6%
Isoperla grammatica	0.1028	0.1044	+1.6%	0.1040	+1.2%
Ecdyonurus picteti	0.1857	0.1863	+0.3%	0.1883	+1.4%
Perla grandis	0.1426	0.1445	+1.3%	0.1449	+1.6%
Rhithrogena alpestris	0.2009	0.2030	+1.0%	0.2059	+2.5%
Ecdyonurus venosus	0.0993	0.1036	+4.3%	0.1021	+2.8%
Ephemera danica	0.0569	0.0606	+6.5%	0.0588	+3.3%
Hydropsyche siltalai	0.1079	0.1152	+6.7%	0.1117	+3.5%
Rhyacophila torrentium	0.3406	0.3541	+4.0%	0.3537	+3.8%
Isoperla rivulorum	0.0831	0.0895	+7.7%	0.0892	+7.3%

Table S5.12: Changes in mean occurrence probability ($\overline{p}$) of species according to scenario RCP8.5 in the Southern Alps. Relative change (%) is calculated with respect to period 2015-2025. Species are ordered according to the relative change in 2080-2090.

**Species**	**2015-2025**	**2055-2065**		**2080-2090**	
	$\overline{p}$	$\overline{p}$	Δ (%)	$\overline{p}$	Δ (%)
Rhithrogena loyolaea	0.1160	0.0757	-34.8%	0.0532	-54.2%
Protonemura brevistyla	0.1097	0.0795	-27.6%	0.0615	-43.9%
Rhithrogena alpestris	0.2055	0.1604	-22.0%	0.1256	-38.9%
Rhithrogena puthzi	0.2418	0.1913	-20.9%	0.1510	-37.6%
Ecdyonurus picteti	0.1880	0.1514	-19.5%	0.1235	-34.3%
Drusus discolor	0.1736	0.1406	-19.0%	0.1152	-33.6%
Protonemura nimborum	0.1039	0.0869	-16.3%	0.0719	-30.8%
Nemoura mortoni	0.5016	0.4476	-10.8%	0.3909	-22.1%
Rhyacophila tristis	0.4175	0.3775	-9.6%	0.3360	-19.5%
Nemoura minima	0.1330	0.1189	-10.6%	0.1076	-19.1%
Rhyacophila torrentium	0.3459	0.3209	-7.2%	0.2910	-15.9%
Epeorus alpicola	0.2083	0.1925	-7.6%	0.1803	-13.5%
Allogamus auricollis	0.4645	0.4353	-6.3%	0.4080	-12.2%
Baetis alpinus	0.9178	0.8929	-2.7%	0.8567	-6.7%
Chloroperla susemicheli	0.1837	0.1769	-3.7%	0.1720	-6.3%
Ecdyonurus helveticus	0.6592	0.6417	-2.6%	0.6185	-6.2%
Halesus digitatus	0.0613	0.0594	-3.0%	0.0588	-4.1%
Protonemura lateralis	0.4805	0.4768	-0.8%	0.4651	-3.2%
Brachyptera risi	0.1688	0.1716	+1.7%	0.1652	-2.1%
Odontocerum albicorne	0.1996	0.1991	-0.3%	0.1982	-0.7%
Amphinemura sulcicollis	0.3045	0.3119	+2.4%	0.3084	+1.3%
Rhithrogena semicolorata	0.4358	0.4478	+2.8%	0.4457	+2.3%
Ecdyonurus venosus	0.1019	0.1039	+1.9%	0.1052	+3.2%
Ecdyonurus torrentis	0.0132	0.0135	+1.9%	0.0140	+5.9%
Isoperla grammatica	0.1029	0.1067	+3.8%	0.1095	+6.4%
Potamophylax cingulatus	0.2724	0.2888	+6.0%	0.2924	+7.3%
Baetis muticus	0.3944	0.4118	+4.4%	0.4239	+7.5%
Baetis rhodani	0.8416	0.8857	+5.2%	0.9097	+8.1%
Capnioneura nemuroides	0.0643	0.0697	+8.4%	0.0698	+8.5%
Habroleptoides confusa	0.1363	0.1422	+4.4%	0.1481	+8.7%
Isoperla rivulorum	0.0847	0.0882	+4.1%	0.0932	+10.0%
Epeorus assimilis	0.3548	0.3832	+8.0%	0.4066	+14.6%
Habrophlebia lauta	0.0245	0.0236	-3.4%	0.0285	+16.2%
Perla grandis	0.1403	0.1550	+10.5%	0.1665	+18.7%
Baetis lutheri	0.0422	0.0487	+15.6%	0.0528	+25.3%
Paraleptophlebia submarginata	0.0515	0.0584	+13.4%	0.0670	+30.1%
Centroptilum luteolum	0.0303	0.0349	+15.4%	0.0413	+36.3%
Philopotamus ludificatus	0.2884	0.3667	+27.1%	0.4409	+52.9%
Serratella ignita	0.1056	0.1350	+27.9%	0.1621	+53.5%
Hydropsyche siltalai	0.1077	0.1441	+33.8%	0.1803	+67.4%
Ephemera danica	0.0563	0.0781	+38.7%	0.1057	+87.9%

Table S5.13: Changes in mean occurrence probability ($\overline{p}$) of species according to scenario RCP2.6 in the Central Western Alps. Relative change (%) is calculated with respect to period 2015-2025. Species are ordered according to the relative change in 2080-2090.

**Species**	**2015-2025**	**2055-2065**		**2080-2090**	
	$\overline{p}$	$\overline{p}$	Δ (%)	$\overline{p}$	Δ (%)
Habrophlebia lauta	0.0414	0.0385	-7.0%	0.0371	-10.4%
Capnioneura nemuroides	0.0640	0.0601	-6.1%	0.0588	-8.3%
Rhithrogena loyolaea	0.3017	0.2825	-6.4%	0.2808	-6.9%
Protonemura brevistyla	0.2412	0.2291	-5.0%	0.2265	-6.1%
Nemoura minima	0.0719	0.0685	-4.8%	0.0689	-4.2%
Allogamus auricollis	0.6086	0.5935	-2.5%	0.5846	-3.9%
Rhithrogena puthzi	0.1796	0.1739	-3.1%	0.1764	-1.8%
Halesus digitatus	0.0724	0.0702	-3.0%	0.0712	-1.7%
Centroptilum luteolum	0.0250	0.0251	+0.3%	0.0246	-1.6%
Serratella ignita	0.0390	0.0391	+0.3%	0.0390	+0.1%
Perla grandis	0.1080	0.1092	+1.1%	0.1082	+0.2%
Brachyptera risi	0.0783	0.0780	-0.4%	0.0785	+0.3%
Odontocerum albicorne	0.0833	0.0833	+0.1%	0.0836	+0.4%
Baetis muticus	0.2272	0.2284	+0.5%	0.2282	+0.5%
Baetis alpinus	0.8949	0.8959	+0.1%	0.9016	+0.8%
Protonemura nimborum	0.1671	0.1669	-0.1%	0.1689	+1.1%
Rhithrogena semicolorata	0.2021	0.2057	+1.8%	0.2051	+1.5%
Ecdyonurus picteti	0.3155	0.3189	+1.1%	0.3213	+1.8%
Ecdyonurus torrentis	0.0159	0.0160	+0.8%	0.0161	+1.8%
Habroleptoides confusa	0.0537	0.0547	+1.9%	0.0546	+1.8%
Isoperla grammatica	0.0310	0.0316	+2.0%	0.0316	+1.8%
Rhithrogena alpestris	0.3184	0.3206	+0.7%	0.3255	+2.2%
Potamophylax cingulatus	0.1756	0.1792	+2.1%	0.1797	+2.4%
Amphinemura sulcicollis	0.0752	0.0765	+1.7%	0.0773	+2.8%
Ephemera danica	0.0267	0.0278	+4.0%	0.0276	+3.2%
Rhyacophila torrentium	0.3481	0.3597	+3.3%	0.3598	+3.4%
Rhyacophila tristis	0.3005	0.3009	+0.1%	0.3106	+3.4%
Baetis lutheri	0.0381	0.0392	+3.0%	0.0395	+3.8%
Isoperla rivulorum	0.1553	0.1585	+2.0%	0.1611	+3.8%
Paraleptophlebia submarginata	0.0323	0.0341	+5.7%	0.0336	+4.0%
Nemoura mortoni	0.6277	0.6411	+2.1%	0.6542	+4.2%
Chloroperla susemicheli	0.2069	0.2127	+2.8%	0.2164	+4.6%
Drusus discolor	0.2534	0.2553	+0.7%	0.2652	+4.6%
Ecdyonurus venosus	0.0496	0.0517	+4.3%	0.0526	+6.0%
Epeorus assimilis	0.0892	0.0940	+5.4%	0.0945	+6.0%
Baetis rhodani	0.5772	0.6079	+5.3%	0.6129	+6.2%
Hydropsyche siltalai	0.0444	0.0475	+7.1%	0.0471	+6.2%
Protonemura lateralis	0.4541	0.4716	+3.9%	0.4850	+6.8%
Ecdyonurus helveticus	0.4646	0.4855	+4.5%	0.4987	+7.3%
Epeorus alpicola	0.2088	0.2154	+3.2%	0.2262	+8.3%
Philopotamus ludificatus	0.1366	0.1509	+10.5%	0.1552	+13.7%

Table S5.14: Changes in mean occurrence probability ($\overline{p}$) of species according to scenario RCP8.5 in the Central Western Alps. Relative change (%) is calculated with respect to period 2015-2025. Species are ordered according to the relative change in 2080-2090.

**Species**	**2015-2025**	**2055-2065**		**2080-2090**	
	$\overline{p}$	$\overline{p}$	Δ (%)	$\overline{p}$	Δ (%)
Rhithrogena loyolaea	0.2983	0.2410	-19.2%	0.1634	-45.2%
Protonemura brevistyla	0.2402	0.1961	-18.4%	0.1393	-42.0%
Rhithrogena puthzi	0.1797	0.1432	-20.3%	0.1083	-39.7%
Rhithrogena alpestris	0.3205	0.2792	-12.9%	0.2367	-26.1%
Protonemura nimborum	0.1674	0.1495	-10.7%	0.1360	-18.8%
Ecdyonurus picteti	0.3170	0.2907	-8.3%	0.2616	-17.5%
Rhyacophila tristis	0.3005	0.2765	-8.0%	0.2564	-14.7%
Allogamus auricollis	0.6056	0.5719	-5.6%	0.5209	-14.0%
Drusus discolor	0.2531	0.2320	-8.3%	0.2184	-13.7%
Habrophlebia lauta	0.0403	0.0376	-6.6%	0.0347	-13.7%
Rhyacophila torrentium	0.3519	0.3314	-5.8%	0.3126	-11.2%
Isoperla rivulorum	0.1561	0.1452	-7.0%	0.1414	-9.4%
Baetis alpinus	0.8946	0.8702	-2.7%	0.8341	-6.8%
Nemoura minima	0.0704	0.0666	-5.3%	0.0664	-5.6%
Nemoura mortoni	0.6288	0.6186	-1.6%	0.6088	-3.2%
Halesus digitatus	0.0714	0.0701	-1.8%	0.0718	+0.6%
Epeorus alpicola	0.2099	0.2105	+0.3%	0.2265	+7.9%
Brachyptera risi	0.0778	0.0827	+6.4%	0.0874	+12.3%
Odontocerum albicorne	0.0827	0.0863	+4.4%	0.0932	+12.6%
Capnioneura nemuroides	0.0615	0.0651	+5.9%	0.0694	+12.8%
Ecdyonurus helveticus	0.4671	0.4883	+4.5%	0.5272	+12.9%
Chloroperla susemicheli	0.2073	0.2176	+4.9%	0.2358	+13.7%
Perla grandis	0.1081	0.1113	+2.9%	0.1240	+14.7%
Protonemura lateralis	0.4551	0.4819	+5.9%	0.5247	+15.3%
Rhithrogena semicolorata	0.2026	0.2173	+7.2%	0.2373	+17.1%
Isoperla grammatica	0.0315	0.0335	+6.3%	0.0373	+18.3%
Ecdyonurus venosus	0.0506	0.0512	+1.3%	0.0613	+21.2%
Ecdyonurus torrentis	0.0157	0.0172	+9.3%	0.0193	+22.5%
Baetis muticus	0.2252	0.2508	+11.4%	0.2773	+23.1%
Habroleptoides confusa	0.0533	0.0578	+8.4%	0.0657	+23.2%
Amphinemura sulcicollis	0.0750	0.0823	+9.6%	0.0939	+25.1%
Potamophylax cingulatus	0.1758	0.1978	+12.5%	0.2260	+28.6%
Baetis rhodani	0.5809	0.6696	+15.3%	0.7683	+32.3%
Baetis lutheri	0.0385	0.0455	+18.0%	0.0520	+35.0%
Centroptilum luteolum	0.0250	0.0289	+15.4%	0.0339	+35.4%
Paraleptophlebia submarginata	0.0322	0.0385	+19.3%	0.0448	+38.9%
Epeorus assimilis	0.0904	0.1065	+17.8%	0.1331	+47.2%
Serratella ignita	0.0409	0.0493	+20.8%	0.0689	+68.8%
Hydropsyche siltalai	0.0460	0.0599	+30.3%	0.0804	+75.0%
Ephemera danica	0.0268	0.0356	+33.1%	0.0475	+77.7%
Philopotamus ludificatus	0.1400	0.1983	+41.6%	0.3043	+117.3%

References

Gupta, H. V., Sorooshian, S. & Yapo, P. O. (1999). Status of automatic calibration for hydrologic models: Comparison with multilevel expert calibration. *Journal of Hydrologic Engineering*, 4 (2):135–143. doi: 10.1061/(asce)1084-0699(1999)4:2(135).
